# The Functional and Palaeoecological Implications of Tooth Morphology and Wear for the Megaherbivorous Dinosaurs from the Dinosaur Park Formation (Upper Campanian) of Alberta, Canada

**DOI:** 10.1371/journal.pone.0098605

**Published:** 2014-06-11

**Authors:** Jordan C. Mallon, Jason S. Anderson

**Affiliations:** 1 Department of Biological Sciences, University of Calgary, Calgary, Alberta, Canada; 2 Department of Comparative Biology & Experimental Medicine, University of Calgary, Calgary, Alberta, Canada; Raymond M. Alf Museum of Paleontology, United States of America

## Abstract

Megaherbivorous dinosaurs were exceptionally diverse on the Late Cretaceous island continent of Laramidia, and a growing body of evidence suggests that this diversity was facilitated by dietary niche partitioning. We test this hypothesis using the fossil megaherbivore assemblage from the Dinosaur Park Formation (upper Campanian) of Alberta as a model. Comparative tooth morphology and wear, including the first use of quantitative dental microwear analysis in the context of Cretaceous palaeosynecology, are used to infer the mechanical properties of the foods these dinosaurs consumed. The phylliform teeth of ankylosaurs were poorly adapted for habitually processing high-fibre plant matter. Nevertheless, ankylosaur diets were likely more varied than traditionally assumed: the relatively large, bladed teeth of nodosaurids would have been better adapted to processing a tougher, more fibrous diet than the smaller, cusp-like teeth of ankylosaurids. Ankylosaur microwear is characterized by a preponderance of pits and scratches, akin to modern mixed feeders, but offers no support for interspecific dietary differences. The shearing tooth batteries of ceratopsids are much better adapted to high-fibre herbivory, attested by their scratch-dominated microwear signature. There is tentative microwear evidence to suggest differences in the feeding habits of centrosaurines and chasmosaurines, but statistical support is not significant. The tooth batteries of hadrosaurids were capable of both shearing and crushing functions, suggestive of a broad dietary range. Their microwear signal overlaps broadly with that of ankylosaurs, and suggests possible dietary differences between hadrosaurines and lambeosaurines. Tooth wear evidence further indicates that all forms considered here exhibited some degree of masticatory propaliny. Our findings reveal that tooth morphology and wear exhibit different, but complimentary, dietary signals that combine to support the hypothesis of dietary niche partitioning. The inferred mechanical and dietary patterns appear constant over the 1.5 Myr timespan of the Dinosaur Park Formation megaherbivore chronofauna, despite continual species turnover.

## Introduction

Megaherbivores (herbivorous species whose adults weigh >1,000 kg) exert a strong influence on the structure and population dynamics of their respective ecosystems via their dominating foraging habits [1]. As such, megaherbivore ecology is a subject of ongoing interest, particularly in light of the unique adaptive mode shared by these animals [2]. Sinclair [3] and colleagues [4] have suggested that, unlike smaller forms, mammalian megaherbivores are limited by dietary resources, rather than predation. Among the evidences for this hypothesis is a demonstration of competitive niche displacement between sympatric megaherbivores [5].

Megaherbivorous dinosaurs were particularly diverse on the Late Cretaceous island continent of Laramidia (sensu Archibald [6]), leading some to speculate that their enduring coexistence was facilitated by dietary niche partitioning imposed by competition for limited resources [7]–[9]. If true, this would suggest common evolutionary and ecological constraints operating in two otherwise very disparate groups. Recent work has sought to examine the question of dietary niche partitioning among Laramidian megaherbivores, using the fossil assemblage of the upper Campanian Dinosaur Park Formation (DPF) of Alberta as a model [10]–[14]. The present study continues in this vein, with insight provided by an examination of unworn tooth morphology, dental macrowear and microwear, which reflect the internal mechanical properties and external physical attributes of the foods that the teeth break down over different time scales [15]. Unworn (preformed) tooth morphology reflects the long-term adaptation of teeth over geological time [16]–[18]. Worn tooth morphology, as visible to the naked eye (macrowear), reflects the influence of food properties on tooth shape over ecological time, which spans the majority of an individual's lifetime [15],[19]. Finally, microscopic tooth wear patterns (microwear) form over a relatively short period of time, spanning just weeks to months [20]. Tooth wear can also provide crucial insight into the jaw mechanics employed to rend different food types [21]–[27]. Thus, these three aspects of tooth maturation provide different, but complementary, information regarding feeding ecology, and are therefore considered here in tandem. With these considerations in mind, we predict that, on the hypothesis of limiting food resources, sympatric megaherbivorous dinosaur species should exhibit differences in tooth morphology and wear that reflect dietary niche partitioning.

### Institutional abbreviations

AMNH, American Museum of Natural History, New York; CMN, Canadian Museum of Nature, Ottawa; FMNH, Field Museum of Natural History, Chicago; NHMUK, Natural History Museum, London; ROM, Royal Ontario Museum, Toronto; TMM, Texas Memorial Museum, Austin; TMP, Royal Tyrrell Museum of Palaeontology, Drumheller, Alberta; UALVP, University of Alberta Laboratory of Vertebrate Palaeontology, Edmonton; USNM, National Museum of Natural History, Washington, D. C.; YPM, Yale Peabody Museum, New Haven.

## Materials and Methods

This study generally focuses on the description of intact dentitions associated with skulls to maximize taxonomic resolution. No permits were required for the described study, which complied with all relevant regulations. The total dataset comprised 76 specimens spanning 16 megaherbivorous dinosaur species from the clades Ankylosauria, Ceratopsidae, and Hadrosauridae, all from the DPF (Table S1). Due to a lack of intact ankylosaur dentitions, we also studied numerous isolated teeth attributable to this taxon to understand the variation present therein. We examined gross tooth morphology and macrowear with the occasional aid of a hand lens and light microscopy to elucidate such details as the nature of the enamel-dentine transition and the presence of secondary enamel ridges.

Descriptions of vertebrate dentitions have traditionally been plagued by a lack of standardized terminology of anatomical notation and orientation. For this reason, we used the dental nomenclature proposed by Smith and Dodson [28]. Teeth in the upper and lower jaws are numbered sequentially, based on their position relative to the first, or mesial-most, tooth. For example, the sixth tooth of the right maxilla would be referred to as ‘RM 6’, whereas the tenth tooth of the left dentary would be ‘LD 10’. In hadrosaurids, where multiple teeth per tooth family usually contribute to the occlusal surface, an additional qualifier is given to differentiate the position of the tooth within the tooth family. For example, a newly occluding tooth in the eighth tooth family of the left maxilla would be referred to as ‘LM 8(1)’, whereas an older, more labially positioned tooth in the nineteenth tooth family of the right dentary might be ‘RD 19(3)’. The parenthetical numeration refers to the position of the tooth within the occlusal surface, with the newest occluding tooth designated as ‘(1)’, and all successively older teeth being numbered as appropriate.

## Dental microwear analysis

The microwear dataset (Table S2; http://doi.org/10.5061/dryad.654sh) was necessarily reduced due to taphonomic effects (see below). It comprised 51 specimens spanning 10 genera from the clades Ankylosauria, Ceratopsidae, and Hadrosauridae. We supplemented the ankylosaur microwear dataset with 15 isolated teeth, identifiable only to the family level, because there were otherwise too few specimens to subject this clade to statistical testing. We did not include the ankylosaurids *Dyoplosaurus* [29], [30] and *Scolosaurus* [31], [32], nor the ceratopsid *Spinops* [33], because their teeth are not preserved. There is also some question as to whether all these animals are originally from the DPF because precise locality data are unavailable.

This study differs from most studies of mammalian microwear in two important ways. First, mammalian dental microwear analysis is typically performed on enamel surfaces [34]; however, because dinosaur enamel is so thin (∼100 µm), it is quickly worn away, exposing the underlying dentine. Green [35] demonstrated that dentine, while softer than enamel, nevertheless preserves a comparable dietary signal. For this reason, we examined dentine microwear. Preliminary investigation confirmed that both mantle dentine and orthodentine are exposed on wear facets [36]. The former is characterized by its resistance to wear, causing it to stand proud of the softer orthodentine, which wears more readily to produce a concavity on the occlusal surface [36]. Despite these mechanical differences, examination of microwear revealed that individual features are continuous across the boundary of these two tissues. Therefore, we did not discriminate between microwear on mantle dentine or orthodentine surfaces. Nevertheless, most examined microwear derived from orthodentine because this comprises the majority of the occlusal surface of teeth (particularly in ceratopsids and hadrosaurids).

Second, whereas studies of mammalian dental microwear typically control for such factors as tooth position, facet type, and degree of wear [37]–[39], this is exceedingly difficult to do with dinosaurs, which generally retain a homodont dentition with continual tooth replacement [40]. Furthermore, dinosaur dentitions are often incomplete and individual teeth are frequently devoid of microwear due to taphonomic alteration. For this reason, we quantified microwear across as many tooth positions as possible and established the microwear signal using mean values for a range of variables (see below). This approach makes maximal use of the information available and is reasonable given that tooth shape does not differ significantly along the tooth row in dinosaurs as it does in mammals, implying that microwear does not vary systematically as a result. This assumption was most recently validated by the work of Williams et al. [27] and Fiorillo [41], who demonstrated a lack of systematic microwear variation along the tooth row in hadrosaurids. We combined microwear information from the right and left sides of the jaws where necessary to obtain a representative sample from along the tooth row. We studied dentary teeth preferentially because these are both commonly preserved and most easily accessible due to the fact that their occlusal surfaces face labially. The occlusal surfaces of maxillary teeth typically face lingually and cannot be readily examined; however, sometimes only maxillary teeth were available for examination and thus were used accordingly. Given that maxillary and dentary tooth rows often closely resemble one another—indeed, their occluding relationship requires this—we do not suspect that the occasional use of maxillary teeth would greatly affect the conclusions of this study. Although we did not test this hypothesis explicitly, it is supported by previous investigations of ceratopsid [42] and hadrosaurid [27] microwear.

For the purposes of microwear analysis, we first examined teeth for the presence of wear facets, identified by their flat, shiny appearance. When conservational considerations allowed, we cleaned the facets using several washes of acetone or alcohol and water, applied gently with a cotton swab until no signs of dust or preservatives remained. We then created molds of the teeth using President regular body polyvinylsiloxane (Coltène/Whaledent), from which we made casts using Epotek 301 two-part epoxy.

We examined dental microwear at 35× magnification using a Nikon SMZ1500 stereo light microscope. We aligned the tooth casts within the field of view so that their apices pointed towards the top (or, if the apex was worn away, we aligned the tooth cervix side-to-side). We illuminated the casts from beneath using refracted light because this produced the best relief for viewing microwear features. Despite the presence of wear facets, many teeth showed no signs of microwear under the microscope. Instead, wear facets often appeared matted or frosted due to post-mortem abrasion or acid etching [43]. We rejected these teeth from further analysis. Real microwear features appeared in regular patterns and were restricted to the occlusal surfaces of the teeth [44]. If there was any doubt about the origin of certain features, we rejected the tooth in question from further analysis. We visualized microwear using the high dynamic range imaging (HDRI) method of Fraser et al. [45], which enhances both the visualization of microwear in print and the repeatability of feature quantification over traditional low magnification approaches (e.g., [46]). We photographed microwear using a Nikon D200 digital SLR camera mounted to the microscope. The final tone mapped images were produced in Photomatix Pro 3 (HDRsoft), loaded into ImageJ 1.43s [47], and each was digitally overlaid with a 0.4×0.4 mm bounding box to constrain feature quantification. Where possible, we used two bounding boxes to capture variation within a single tooth facet; however, we took care to ensure independence by making certain that microwear features did not cross through more than one box. We digitally measured the length, width, and orientation of all features passing through each bounding box. From these measurements, we calculated the number of scratches (features four times longer than wide), number of pits (features less than four times longer than wide), and average feature width, for use in subsequent analyses (see below). We selected these variables because they have been shown to discriminate various modern taxa with different diets [46], [48]–[50].

### Jaw mechanics

In addition to the consideration of dental macrowear, we studied preferred jaw movements with the aid of rose diagrams depicting the distributions of microwear scratch length and orientation. We binned scratch lengths in 250 µm increments and scratch angles in 10° increments. To facilitate comparison, we reflected all rose diagrams as appropriate to correspond to teeth from the right dentary. In this way, we calculated scratch angles from left dentary and right maxillary teeth as 180-θ. Scratch angles from the left maxilla did not require correction. All rose diagrams herein depict the mesial direction at 0° and the apical (dorsal) direction at 90°. We created all rose diagrams using Oriana 3.13 (Kovach Computing Services).

### Quantitative microwear comparisons

We compared scratch number, pit number, and average feature width across various taxa. We drew comparisons at coarse (family/suborder), medium (subfamily/family), and fine (genus) taxonomic scales. We did not consider the species level because sample size was consistently too low at this resolution to permit meaningful statistical comparisons (even so, many of the genera considered here are monospecific). We used non-parametric statistics because sample sizes were generally quite small (n≤20). These tests lack the power of parametric statistics but are more robust against committing Type I errors (reporting differences where none exist). When testing for differences between the medians of two groups of univariate microwear data, we used the Mann-Whitney U test, and the Kruskal-Wallis test to compare the medians of more than two groups. When comparing two or more groups of multivariate data, we used non-parametric multivariate analysis of variation (NPMANOVA), which tests for differences using a specified distance measure [51]. We used the Mahalanobis distance measure [52] because it is better suited to non-spherically symmetric data than the traditional Euclidean distance measure. In NPMANOVA, significance is estimated by permutation across groups, which we performed using 10,000 replicates. We set statistical significance for all tests at α = 0.05.

Where appropriate, we conducted post-hoc pairwise comparisons with Bonferroni correction. Bonferroni correction was designed to counteract the problem of multiple comparisons, whereby the probability of committing a type I error increases with the number of simultaneous comparisons being made [53]. This problem is rectified by multiplying the *p*-value by the number of pairwise comparisons, effectively lowering the significance level. However, because Bonferroni correction provides little power and is probably too conservative [53], [54], we also report uncorrected probabilities for interpretation.

Because the DPF does not represent a single assemblage of contemporaneous organisms, time-averaging is an issue. This has the effect of masking palaeoecological patterns that are otherwise distinguishable only at fine temporal resolutions [55]. For this reason, we minimized the effects of time-averaging by making the above comparisons within each of the two most inclusive Megaherbivore Assemblage Zones (MAZs) identified by Mallon et al. [10]. To summarize, MAZ-1 encompasses the lower 28 m of the DPF, whereas MAZ-2 encompasses intervals from 29–52 m. While this time-constrained approach theoretically increases the probability of recovering differences that would otherwise be masked by the effects of time-averaging, there is a trade-off in that sample size (and hence statistical power) is reduced considerably. Also, this approach does not completely remove the effects of time-averaging because the abovementioned MAZs are themselves time-averaged over a period of approximately 600 Kyr [10].

Finally, to help visualize microwear relationships within and between MAZs, we used principal component analysis (PCA). This is an ordination technique that allows the projection of a multivariate dataset down to a few orthogonal dimensions of maximal variance (principal components or PCs) to simplify interpretation of the data distribution [56]. PCA returns both a series of eigenvalues that indicates the amount of variation explained by each axis, and a set of loadings that denotes the importance of each variable in contributing to the data spread along each axis (depicted here as vectors in microwear space). We performed PCA on the correlation matrix because not all measurements were of the same scale [56]. All statistical and ordination procedures were performed using the software program PAST 2.12 [57].

## Results

### *Ankylosauria*

#### Unworn tooth morphology

**Figure 1:**
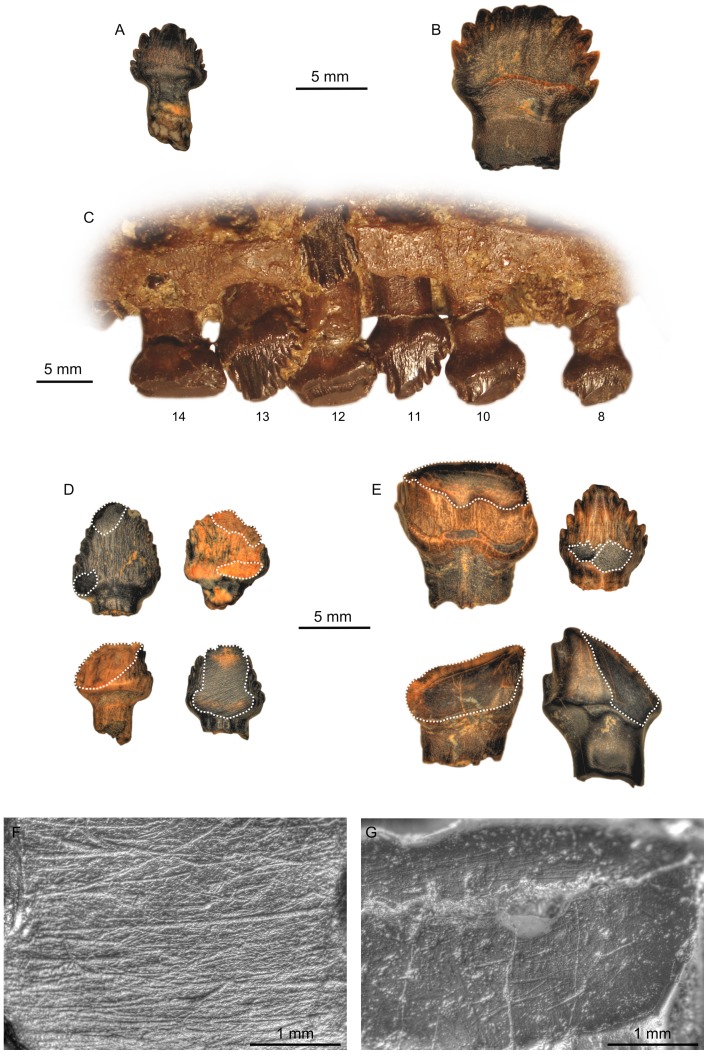
Overview of ankylosaur teeth. A, unworn ankylosaurid tooth (TMP 1992.036.1178) in lingual view; B, unworn nodosaurid tooth (TMP 2000.012.0024) in lingual view; C, partial left maxillary tooth row of *Panoplosaurus mirus* (ROM 1215) in lingual view, exemplifying the distal shift in both tooth size and wear facet orientation (tooth positions numbered); D, isolated ankylosaurid teeth exhibiting various states of wear (facets shown with dashed outline). Clockwise from top left: TMP 1997.016.0106, TMP 1991.036.0734; TMP 1997.012.0042, TMP 1989.050.0026; E, isolated nodosaurid teeth exhibiting various states of wear (facets shown with dashed outline). Clockwise from top left: TMP 2000.012.0027, TMP 1997.012.0005 (this tooth exhibits paired, mesiodistally arranged wear facets indicative of interlocking tooth occlusion), TMP 1994.094.0014, TMP 1992.036.0101; F, microwear from an isolated ankylosaurid tooth (TMP 1991.050.0014) exhibiting many mesiodistally oriented scratches; G, pitted and scratched LM 12 microwear of the nodosaurid *P. mirus* (ROM 1215).

Ankylosaur teeth (Figure 1) bear straight roots and labiolingually compressed, phylliform (leaf-shaped) crowns with a distally-offset apex and apical denticulate carinae [58]–[61]. In the ankylosaurid *Euoplocephalus tutus*, unworn tooth crowns (Figure 1A) are pointed and small, rarely exceeding 7 mm in apicobasal height and 6 mm in mesiodistal width (height:width ratio  = 1.17). The number of denticles in isolated ankylosaurid teeth range from 0.83–1.38 denticles/mm. The labial and lingual surfaces of the crown are fluted apicobasally, but the fissures do not coincide with the notches between the marginal denticles [59], [60], [62]. By contrast, in the nodosaurid *Panoplosaurus mirus*, unworn tooth crowns (Figure 1B) regularly approach 12 mm in apicobasal height and 11 mm in mesiodistal width (height:width ratio  = 1.09), are less tapered apically, and the labial and lingual surfaces bear fluting that is coincident with the apical denticle notches [59], [60]. The number of denticles in isolated nodosaurid teeth ranges from 0.56–0.73 denticles/mm. Nodosaurid tooth crowns also possess a conspicuous basal cingulum that is better developed on the labial side of both the maxillary and dentary teeth [59]–[60].

Ankylosaur teeth are typically set in short (<150 mm), medially bowed tooth rows. The maxillary tooth row is concave up in lateral aspect, and the dentary tooth row is correspondingly convex up. The curvature of the tooth rows is more exaggerated in *Euoplocephalus tutus* than in *Panoplosaurus mirus*. Tooth size generally increases distally along the tooth row (Figure 1C), although intact dentitions are rarely preserved. One replacement tooth is commonly present in each tooth position. In *E. tutus*, there are up to 24 maxillary teeth and 21 dentary teeth. Tooth counts are lower and more asymmetrical in *P. mirus* (ROM 1215), which possesses 17 maxillary teeth and 11 dentary teeth [63]; however, in another specimen (TMP 1998.098.0001) there are 17 maxillary teeth and 19 dentary teeth. These differences may ultimately prove to be of taxonomic significance, but that is beyond the scope of this study. The lower tooth count in *P. mirus* relative to *E. tutus* is likely owed to the larger size of the teeth in nodosaurids.

#### Dental macrowear

Ankylosaur teeth are commonly worn (contra Hwang [64]), and wear facets are oriented variably across the crown surfaces [59] (Figure 1D, E). Barrett [65] noted that ankylosaur teeth typically bear oblique wear facets that occur either individually on the labial (or lingual) crown face, or as paired surfaces that develop along the carina on the mesial and distal sides of the apex as opposing teeth interlock. Rybczynski and Vickaryous [66] emphasized that ankylosaur teeth only rarely show paired wear facets, and that single facets dominate, implying that ankylosaur teeth did not typically interlock. The teeth of *Euoplocephalus tutus* in particular were said to consistently bear single, vertical facets continuous across adjacent teeth.

To determine the relative incidence of paired versus single wear facets on ankylosaur teeth, we examined a sample of 100 isolated ankylosaur teeth from the DPF (19 ankylosaurid; 81 nodosaurid) for the presence of paired facets (Table S3). Of the 19 ankylosaurid teeth, seven (37%) possess two wear facets. However, these facets either occur on opposite sides of the tooth or are arranged apicobasally (e.g., Figure 1D). In no case are the paired facets arranged mesiodistally, as would be expected if opposing teeth interlocked. Thirteen of the 19 (68%) isolated ankylosaurid teeth bear vertical wear facets; the remainder possess oblique facets only. Nine of the 81 (11%) isolated nodosaurid teeth possess more than one wear facet; however, only in two cases (2%) do paired, mesiodistally arranged wear facets occur on the same surface of the tooth. Twenty-four of the 81 (30%) nodosaurid teeth bear vertical facets, whereas the rest bear either horizontal or oblique facets only.

Although rare, intact ankylosaur dentitions corroborate the general observations above. The worn maxillary teeth of *Euoplocephalus tutus* (AMNH 5405) bear single, vertical or sub-vertical wear facets on their lingual surfaces. Facets on adjacent teeth from the middle of the tooth row appear coplanar [66]; however, whether the same is true of teeth towards either end of the tooth row is difficult to determine because the more mesially positioned teeth have rotated within their sockets, whereas the more distally positioned teeth are broken or missing.

The maxillary teeth of *Panoplosaurus mirus* (ROM 1215) likewise bear a single wear facet on their lingual surface, but these exhibit an interesting pattern whereby the facets shift from a sub-vertical to horizontal inclination distally along the tooth row ([59]: fig. 20.1, [63], [66]), (Figure 1C). The most distal teeth are worn down to the cingulum. Whether the facets of adjacent teeth are coplanar as in *Euoplocephalus tutus* cannot be determined because alternate teeth are either missing or not in occlusion. The sixth maxillary tooth deviates from the pattern just described in bearing a single, large facet on its labial (rather than lingual) surface. Teeth from a second specimen of *P. mirus* (TMP 1998.098.0001) appear to exhibit the same distal shift from sub-vertically to horizontally inclined wear facets, but are generally too poorly preserved to be certain.

#### Dental microwear

**Figure 2:**
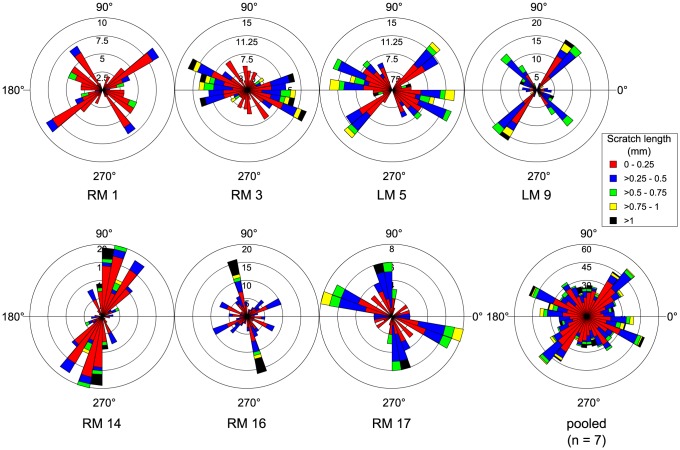
Rose diagrams depicting the orientation of microwear scratches along the tooth row of *Euoplocephalus tutus* (AMNH 5405). Where possible, scratch orientations have been standardized to correspond to teeth from the right dentary (0° =  mesial, 90° =  apical).

**Figure 3:**
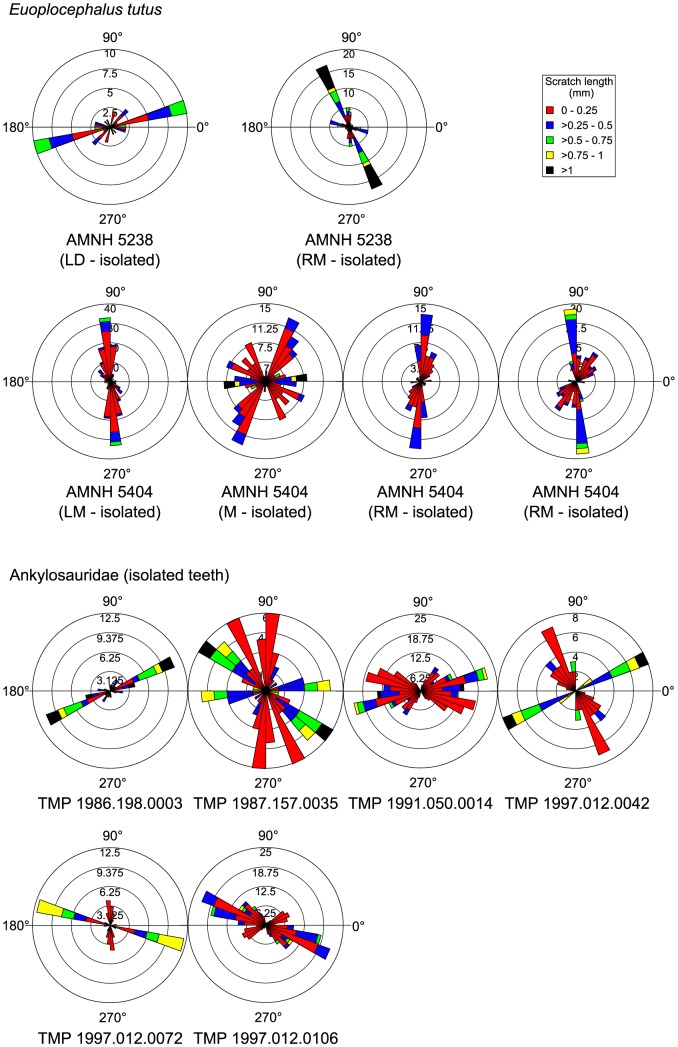
Rose diagrams depicting the orientation of microwear scratches in isolated teeth of *Euoplocephalus tutus* and other unidentified ankylosaurids. Note the dominance of mesiodistally oriented scratches, particularly on the unidentified teeth. Where possible, scratch orientations have been standardized to correspond to teeth from the right dentary (0° =  mesial, 90° =  apical).

Ankylosaur teeth regularly exhibit microwear (Figure 1F, G), but the paucity of intact ankylosaur dentitions makes systematic study of microwear along their jaws difficult. The best available ankylosaur dentition from the DPF belongs to *Euoplocephalus tutus* (AMNH 5405). We recovered microwear from seven maxillary teeth from this specimen, representing various points along the length of the tooth row (Figure 2). Individual teeth are characterized by bi- or polymodal scratch distributions, but the signal is highly variable along the length of the jaw and does not appear to follow any discernible pattern; therefore, to facilitate interpretation, we pooled the scratch data for all teeth into a single rose diagram (Figure 2). A few features are notable. First, there is a distinct mode of scratch orientations from 40°–60°, comprising mostly short scratches (<0.50 mm). Second, there is another, albeit less distinct, mode of low angled scratches that trend dorsodistally-ventromesially (150°–160°), comprising a slightly greater proportion of longer scratches. Finally, the majority of the longest scratches (>1 mm) are oriented apicobasally (80°–110°), but these do not comprise a distinct mode. A Spearman's rank order correlation test reveals no significant correlation between tooth position and either scratch number (n = 7, ρ = −0.143, *p*>0.05), pit number (n = 7, ρ = −0.450, *p*>0.05), or average feature width (n = 7, ρ = 0.536, *p*>0.05; Table S4). Unfortunately, we were unable to locate other intact *E. tutus* dentitions for examination, but the wear fabrics present on isolated ankylosaurid teeth attributable to this genus generally corroborate the patterns identified here, particularly the prominence of mesiodistally oriented scratches. However, four isolated teeth from AMNH 5404 show a distribution wherein most scratches, usually the longest, are oriented apicobasally (Figure 3).

**Figure 4:**
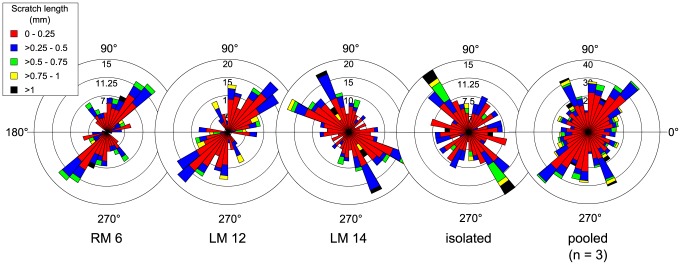
Rose diagrams depicting the orientation of microwear scratches along the tooth row of *Panoplosaurus mirus* (ROM 1215). The pooled sample does not include the isolated tooth because it could not be placed confidently within the jaw. Where possible, scratch orientations have been standardized to correspond to teeth from the right dentary (0° =  mesial, 90° =  apical).

**Figure 5:**
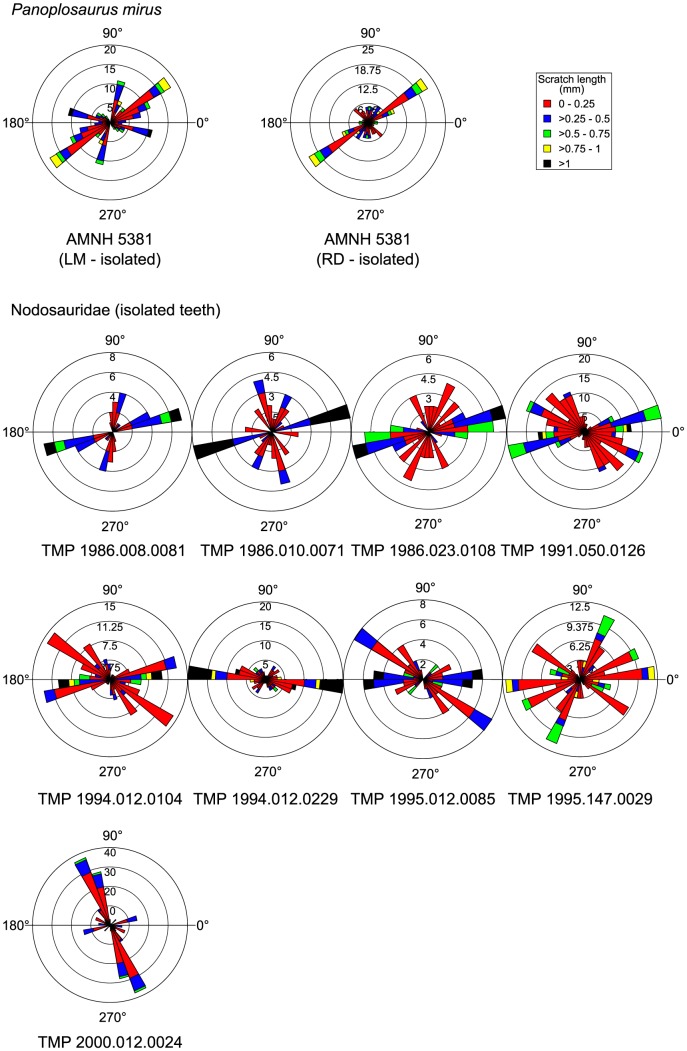
Rose diagrams depicting the orientation of microwear scratches in isolated teeth of *Panoplosaurus mirus* and other unidentified nodosaurids. Most of these teeth are dominated by mesiodistally oriented scratches, as in ankylosaurids. Where possible, scratch orientations have been standardized to correspond to teeth from the right dentary (0° =  mesial, 90° =  apical).

The best available *Panoplosaurus mirus* dentition pertains to ROM 1215. We were able to obtain microwear data from just three tooth positions along the caudal half of the maxilla, plus an isolated dentary tooth (Figure 4). The microwear signal along the tooth row does not appear as variable as in *Euoplocephalus tutus*, although this may simply reflect the fact that fewer teeth are available from ROM 1215. Two of the teeth (RM 6 and LM 12) show unimodal scratch orientations with dorsomesially-ventrodistally inclined (30°–60°) scratches, most of which tend to be among the longest (>0.5 mm). The isolated dentary tooth shows a similar distribution of scratches, but it is not possible to comment on their specific orientation because the position of the tooth is unknown. A single tooth (LM 14) shows primarily dorsocaudally oriented striae (100°–160°), encompassing the longest features (>0.75 mm). Pit percentage increases distally, from 7% to 17%, but sample size is too small to permit statistical testing of its significance. Nevertheless, this distal increase in pit percentage accords with the distal shift from subvertical, shearing wear facets to subhorizontal, crushing wear facets. Other isolated nodosaurid teeth attributable to *P. mirus* support the general patterns described here (Figure 5). Additionally, they exhibit a preponderance of mesiodistally oriented scratches, as in *E. tutus*.

### *Ceratopsidae*

#### Unworn tooth morphology

**Figure 6:**
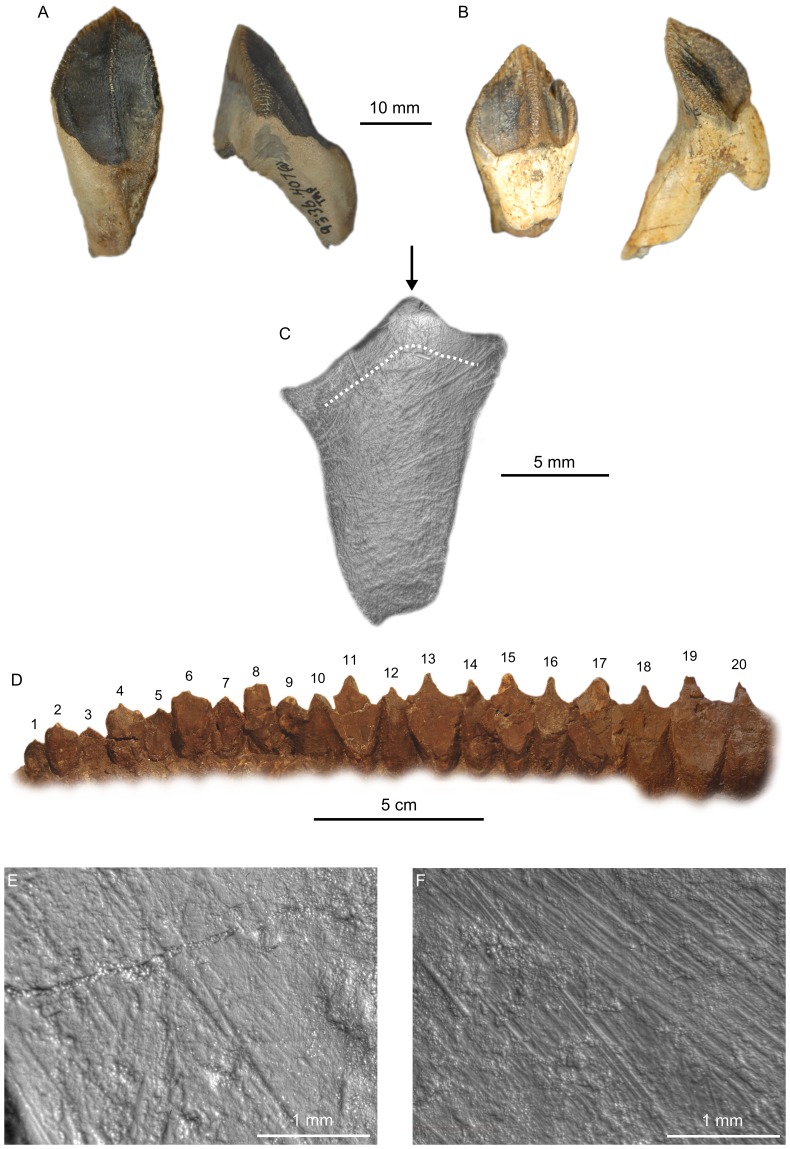
Overview of ceratopsid teeth. A, isolated right maxillary tooth (TMP 1993.036.0407) in lingual (left) and mesial (right) views; B, isolated left dentary tooth (TMP 2002.012.0054) in labial (left) and distal (right) views; C, isolated right dentary tooth (AMNH 21606) in occlusal view. Arrow denotes tooth apex. Dashed line indicates boundary between mantle dentine (external) and orthodentine (internal); D, left dentary tooth row of *Centrosaurus apertus* (ROM 767) in labial view, showing the first 20 teeth (numbered); E, RD 17 microwear of the centrosaurine *Ce. apertus* (ROM 767); F, LD 22 microwear of the chasmosaurine *Chasmosaurus* sp. (ROM 839).

Ceratopsid teeth are highly derived with respect to the phylliform condition of more basal ornithischians, but there is little variation in dental morphology within the clade. The gently curved teeth taper to a point apically, and their bifurcated roots are arranged labially and lingually. The tooth crowns are capped by enamel on only one side; lingually on the dentary teeth, and labially on the maxillary teeth (Figure 6A, B). A pronounced, crenulated carina (primary ridge) bisects the enamel cap apicobasally. The ridge is low and offset distally on the maxillary teeth; it is much more prominent and offset mesially on the dentary teeth. Some specimens (e.g., *Centrosaurus apertus*, ROM 767; *Chasmosaurus russelli*, TMP 1981.019.0175; *Vagaceratops irvinensis*, CMN 41357) occasionally possess one or two subsidiary ridges on either side of the primary ridge, but these do not appear to vary systematically. The margins of the enamel cap bear small, tightly spaced denticles (1.04–1.26 denticles/mm).

**Figure 7:**
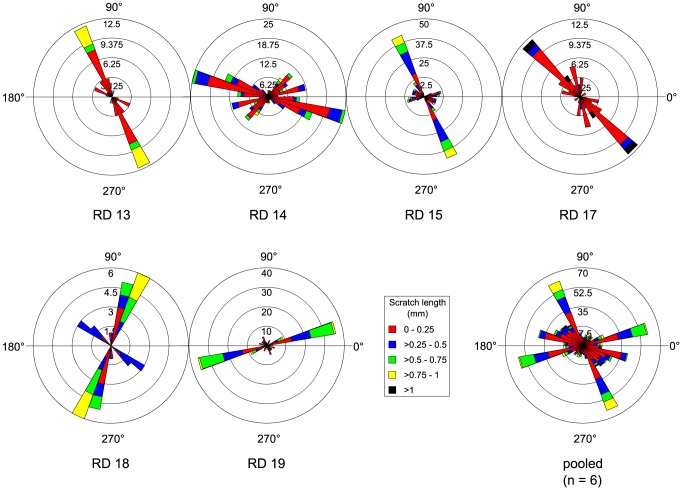
Rose diagrams depicting the orientation of microwear scratches along the tooth row of *Centrosaurus apertus* (ROM 767). Where possible, scratch orientations have been standardized to correspond to teeth from the right dentary (0° =  mesial, 90° =  apical).

Ceratopsid teeth occur in compact dental batteries unlike anything seen in any living animal (Figure 6C). The teeth are arranged in vertical columns (tooth families) that repeat for approximately half the length of the mandible. The tooth rows of all species examined here reach a similar adult size and do not exceed 350 mm in length (*Centrosaurus apertus*, TMP 1997.085.0001). They are very slightly bowed medially and approach one another rostrally ([67]:fig. 7). There are no more than 29 tooth families present within a single tooth-bearing element from the DPF sample (*C. apertus*, ROM 767, TMP 1997.085.0001), but up to 40 tooth families have been reported in *Triceratops* [68]. Each tooth family may contain up to four or five successional teeth, with smaller tooth families occurring nearer the mesial and distal extremities of the tooth row. Within each tooth family, the teeth are stacked so that the bifurcated root of one tooth straddles the crown of that succeeding it. This results in a strong and well-integrated system that ensured the continual replacement of teeth without gaps, although tooth staggering along the leading edge of the occlusal surface occurs in some specimens (e.g., *C. apertus*, AMNH 5351). Edmund [69] noted that some specimens preserve small masses of spongy bone that overlap the crowns and roots of successional teeth, which may have prevented the premature loss of roots as the teeth became worn. This spongy bone is likely cementum [36], [68] covering the shorter, labial root of the overlying dentary tooth.

#### Dental macrowear

Only the apicalmost tooth within a single tooth family enters into occlusion. As the teeth occlude, they develop vertical wear facets, creating a continuous shearing surface along the length of the tooth row. The teeth are arranged within the jaw so that the enamelled tooth carinae form a serrated pattern along the leading edge of the shearing surface (“mega-serrations” of Ostrom ([70]:p. 295); Figure 6C). Individual tooth facets are roughly triangular in outline and reveal the inner histology of the tooth (Figure 6D). The apical margin of the tooth is capped by a thin (∼100 µm) enamel layer externally. The underlying dentine is divided into superficial mantle dentine, ∼1 mm thick, that thins to ∼200 µm mesially and distally, beneath which the orthodentine forms the majority of the facet.

#### Dental microwear

Ceratopsid teeth are regularly worn, but dental microwear is comparatively difficult to find (Figure 6E, F). The best ceratopsid specimen available for dental microwear analysis belongs to *Centrosaurus apertus* (ROM 767). Six teeth from the middle of the dentary tooth row preserve discernible microwear. Scratches from individual teeth typically exhibit uni- or bimodal distributions, but the pooled data support the existence of a polymodal scratch distribution along the length of the tooth row (Figure 7). Two modes are particularly prominent: a steeply inclined mode trending dorsodistally-ventromesially (110°–120°, 290°–300°), and a second, shallowly inclined mode trending strongly mesiodistally (10°–20°, 190°–200°). Both modes comprise comparable numbers of scratches, but the first mode includes proportionately longer scratches (> 750 µm). A third mode also comprises mesiodistally trending scratches (160°–170°, 340°–350°), but these are neither as numerous nor as long as in the other two modes. Spearman's rank-order correlation tests demonstrate no significant correlation between tooth position and either scratch count (n = 6, ρ = −0.257, *p*>0.05), pit count (n = 6, ρ = 0.667, *p*>0.05), or average feature width (n = 6, ρ = 0.143, *p*>0.05; Table S5).

**Figure 8:**
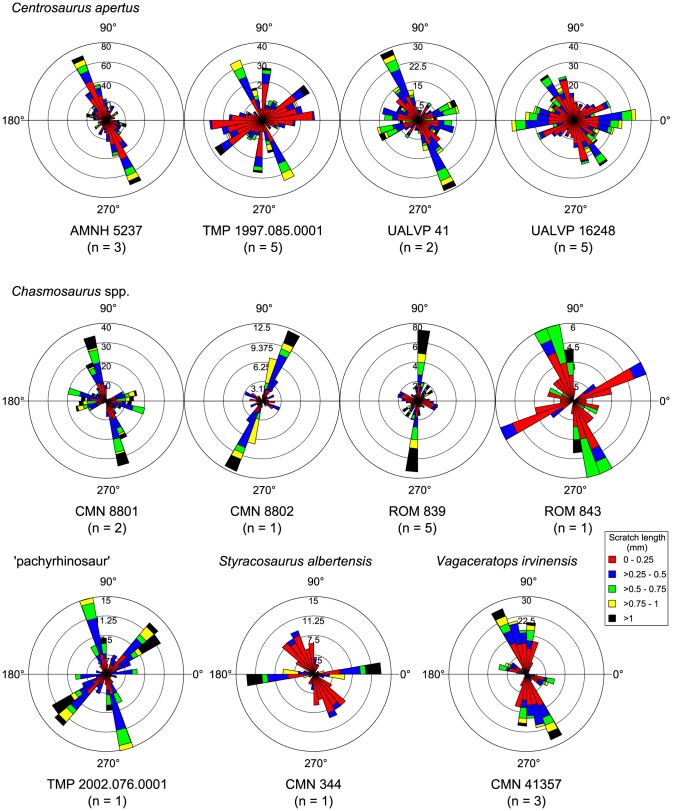
Rose diagrams depicting the orientation of microwear scratches in ceratopsids. Most scratches are oriented dorsodistally-ventromesially; however, there appears to be a second common mode whereby the scratches are oriented mesiodistally. Where possible, scratch orientations have been standardized to correspond to teeth from the right dentary (0° =  mesial, 90° =  apical).

Other ceratopsid dentitions exhibit similar polymodal microwear patterns, albeit with some variation in the length and orientations of the scratches that define them (Figure 8). Most specimens (*Centrosaurus apertus*, AMNH 5237, TMP 1997.085.0001, UALVP 41, 16248; *Chasmosaurus belli*, ROM 843; *Ch.* sp., CMN 8801, ROM 839; *Styracosaurus albertensis*, CMN 344; *Vagaceratops irvinensis*, CMN 41357) possess a distinct mode of apicobasally to dorsodistally-ventromesially inclined scratches, ranging between 100° and 130° (or 280° and 310°). This mode is usually dominant, comprising the most numerous and longest scratches (>1 mm). Many specimens (*Ce. apertus*, TMP 1997.085.0001, UALVP 41, 16248, *Ch. belli*, ROM 843; *Ch.* sp., CMN 8801, ROM 839; *S. albertensis*, CMN 344; *V. irvinensis*, CMN 41357) also exhibit a distinct mode of mesiodistally trending scratches (0°–30°, 180°–210°; 150°–180°, 330°–360°) that may occasionally eclipse other modes in having longer and more numerous scratches. Finally, some specimens (*Ce. apertus*, CMN 8798; *Ch.* sp., ROM 839; ‘pachyrhinosaur’, TMP 2002.076.0001) exhibit a conspicuous mode of dorsomesially-ventrodistally inclined scratches (40°–90°, 220°–270°), which may be quite long (>1 mm), but this mode occurs only rarely.

### *Hadrosauridae*

#### Unworn tooth morphology

**Figure 9:**
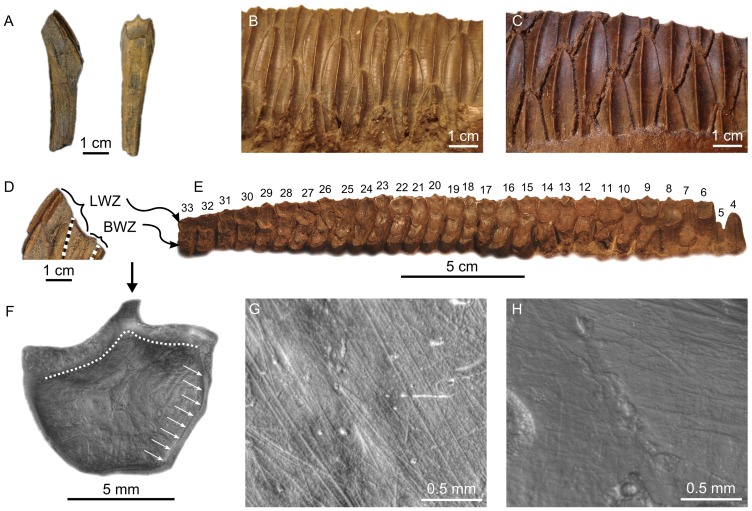
Overview of hadrosaurid teeth. A, isolated dentary tooth (TMP 1966.031.0032) in unknown (mesial or distal) view (left), and labial view (right); B, left dentary tooth battery of the hadrosaurine *Gryposaurus notabilis* (ROM 873) in lingual view; C, right dentary tooth battery of the lambeosaurine *Lambeosaurus lambei* (CMN 351) in lingual view; D, transverse section of a hadrosaurid dentary tooth battery (TMP 1966.031.0032), illustrating the lingual worn zone (LWZ) and buccal worn zone (BWZ). Dashed lines denote tooth contacts; E, right dentary tooth battery of *L. lambei* (CMN 351) in labial view, showing the occlusal surface (tooth positions numbered); F, right dentary tooth 12(1) of *Corythosaurus* sp. (ROM 1947) in occlusal view. Large, black arrow denotes tooth apex. Dashed line indicates boundary between mantle dentine (external) and orthodentine (internal). Small, white arrows point to stepped orthodentine-coronal cementum interface, which can be used to infer the feeding movements of the mandible; G, RD 13(1) microwear of *Prosaurolophus maximus* (CMN 2870); H, LD 24(2) microwear of *Corythosaurus* sp. (ROM 868).

Hadrosaurid teeth are lanceolate, and the crown is offset at an angle from the root (Figure 9A). In lambeosaurines, the angle is >145°, whereas in hadrosaurines, the angle ranges from 120°–140° [71], [72]. As in ceratopsids, the tooth crowns are capped by enamel on only one side; maxillary tooth crowns bear enamel on their labial surfaces, whereas those of the dentary bear enamel on their lingual surfaces. The tooth crowns of all hadrosaurids are diamond-shaped, but lambeosaurines purportedly have mesiodistally narrower tooth crowns than hadrosaurines [72] (Figure 9B, C). While this appears to be generally true, average crown height:width ratios (H:W) do not discriminate these clades perfectly. For example, the H:W of *Corythosaurus casuarius* ROM 1933 (a lambeosaurine) is 2.17 (n = 10), which is equal to that of a large dentary plausibly attributable to the hadrosaurine *Prosaurolophus maximus* (CMN 8894; n = 13). Likewise, the H:W of *Gryposaurus notabilis* ROM 873 (a hadrosaurine) is 2.74 (n = 2). The enamel cap of the hadrosaurid tooth crown is bisected by a smooth median carina. This is reportedly straight in hadrosaurines and sinusoidal in lambeosaurines [72], but exceptions are again common. For instance, in CMN 2277 (*P. maximus*), the mesial dentary teeth bear sinusoidal carinae, whereas the carinae are weakly sinusoidal to straight in CMN 8633 (*Lambeosaurus* sp.), ROM 1933 (*C. casuarius*), and CMN 2869 (*L*. *lambei*). Lambeosaurine teeth may also bear one or two subsidiary ridges on either side of the median carina [72], but the exact number does not appear to vary systematically. For example, both *C. casuarius* (ROM 1933) and *L. lambei* (CMN 2869) possess two subsidiary ridges on either side of the carina. However, another specimen of *L. lambei* (CMN 351) possesses only a single subsidiary ridge on either side of the carina. In all hadrosaurid teeth, the margins of the enamel cap are denticulate, but the denticles are typically coarser in lambeosaurines [72].

Hadrosaurid teeth are arranged in impressive dental batteries (Figure 9B–D), qualitatively similar to those of ceratopsids, but differing in several ways. Hadrosaurid dental batteries are more compact due to their narrower teeth, having ∼15 more tooth families within a tooth row of equal length. Lambeosaurine tooth rows do not exceed 320 mm in length (*Lambeosaurus clavinitialis*, TMP 1981.037.0001), but those of the larger hadrosaurines *Gryposaurus notabilis* and *Prosaurolophus maximus* may approach 400 mm. Hadrosaurid maxillae usually possess approximately five more tooth families than the corresponding dentaries, but the maxillae have fewer teeth within each tooth family [73]. Hadrosaurine jaws reportedly contain more tooth families than those of lambeosaurines, probably as a result of the larger body sizes observed in the former taxon [12], [72]. For example, whereas the dentary of *L. clavinitialis* (TMP 1981.037.0001) may contain up to 43 tooth families, that of *P. maximus* (TMP 1984.001.0001) exceeds 47. Also in contrast to the condition in ceratopsids, each tooth family contains up to six successional teeth [73], [74], with larger tooth families occurring nearer the centre of the jaw. Teeth vary in size and shape throughout the jaw such that those present in the middle of the jaw tend to be taller and narrower than those further mesially and distally.

#### Dental macrowear

As in ceratopsids, hadrosaurid dental batteries develop continuous wear facets along the length of the tooth row; however, they are unique in that more than one tooth per tooth family may contribute to the occlusal surface. In the maxilla, up to two teeth per tooth family are in occlusion, whereas up to four teeth per tooth family may contribute to the occlusal surface in the dentary (Figure 9D). For this reason, the wear surface of the hadrosaurid dental battery has been compared to a tessellated pavement [74]. The wear surface is gently sinusoidal and more obliquely inclined than in ceratopsids. Wear surface angulation typically varies along the tooth row, resulting in an undulating occlusal plane. The wear surface is oriented ∼40° from horizontal in the middle of the tooth row, and approaches 55° mesially and distally. Weishampel [26] stated that the wear surface of *Prosaurolophus maximus* is more steeply inclined (50°–60°), but we could not verify this. The occlusal surface of the dentary tooth row is more concave than that of the maxilla. This concavity gives rise to two distinct planar surfaces along the tooth row, offset between 125° and 168° (Figure 9E). The transition between these two surfaces may be either sharp or graded, and has been described as a “longitudinal groove” ([26]:p. 62). Wear facets on the labial side of the groove comprise the buccal worn zone (BWZ), whereas those on the lingual side comprise the lingual worn zone (LWZ) [26].

As exposed on the occlusal surface of the hadrosaurid dentition, individual teeth are approximately U-shaped in cross-section with a labially (or lingually) projecting median carina (Figure 9F). The superficial enamel layer exposed on the occlusal surface is ∼100 µm thick. The underlying mantle dentine measures up to ∼700 µm thick, but the majority of the facet area is composed of orthodentine. Wear facets are shallowly concave where the soft orthodentine has undergone increased erosion relative to the harder mantle dentine and enamel. In some specimens with well-preserved occlusal surfaces of the dentary teeth (e.g., *Corythosaurus casuarius*, ROM 1933; *C. intermedius*, TMP 1982.037.0001, 1992.036.0250; *C.* sp., ROM 1947; *Lambeosaurus clavinitialis*, CMN 8703), the orthodentine is most heavily excavated along the mesiolingual edges of individual tooth facets where it abuts the harder enamel of adjacent teeth.

#### Dental microwear

Some authors [26], [75] have questioned whether teeth in the BZW of the hadrosaurid dentition were functional, suggesting that they may instead represent the remains of once functional teeth that have since migrated lingually out of occlusion. We examined dental microwear (Figure 9G, H) to test this hypothesis. Assuming teeth in the BWZ were functional, they would likely have served either a crushing or grinding function, given the shallower attitude of their occlusal surfaces. If used for crushing, we would predict that the tooth facets would possess a higher incidence of microwear pitting relative to their adjacent counterparts in the LWZ, as the mandible moved in an orthal fashion. If used for grinding, we would predict that the facets would exhibit a higher incidence of low-angled scratches oriented mesiodistally relative to their adjacent counterparts in the LWZ, as the mandible moved in a propalinal fashion. In the latter scenario, we would also predict that scratch orientation in the BWZ (measured as r, the mean vector length [76]) should be less variable than in the LWZ because relative jaw movement would be restricted to a single plane. If teeth in the BWZ were functionless (i.e., non-occluding), their facets would be expected to preserve a microwear signal similar to those located in the LWZ.

Dental microwear is difficult to find in the BWZ because most teeth in this zone are highly abraded. This may support the null hypothesis because, if these teeth were non-functional, we would expect them to become abraded by foodstuffs held in the jaws; we would not expect these teeth to show signs of attrition if they did not occlude with corresponding teeth in the maxilla. However, because teeth in the BWZ are so heavily worn, they are very small and possess correspondingly small occlusal surfaces. Therefore, the general lack of microwear on these teeth might simply reflect a lack of surface area available for study; after all, the vast majority of teeth examined in this study, regardless of where they are located in the dentition, are abraded, likely post-mortem. It is therefore difficult to determine the significance of tooth abrasion in the BWZ.

**Figure 10:**
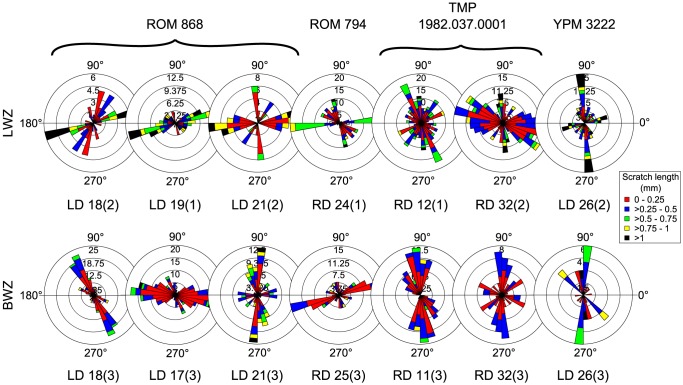
Comparison of microwear scratch orientation between the lingual worn zone (LWZ) and buccal worn zone (BWZ) of the dentary tooth battery. Note that scratches in the BWZ are not more mesiodistally inclined than in the LWZ, as would be expected if teeth in the BWZ served a grinding function. Where possible, scratch orientations have been standardized to correspond to teeth from the right dentary (0° =  mesial, 90° =  apical).

We found only seven cases of microwear on teeth in the BWZ that can be compared to microwear from adjacent teeth in the LWZ (it was occasionally necessary to compare teeth between neighbouring tooth families; Table S6). A Mann-Whitney U test demonstrates that arcsine-transformed pit percentage does not differ significantly between teeth from different zones (n = 14, U = 20.5, *p*>0.05), suggesting that teeth in the BWZ were probably not employed for crushing. Likewise, there is no evidence to suggest that teeth in the BWZ served a grinding function. Scratches on teeth from the BWZ are not more mesiodistally inclined than those from the LWZ (Figure 10), and there are no significant differences in r-values between teeth from the two zones (n = 14, U = 17, *p*>0.05).

**Figure 11:**
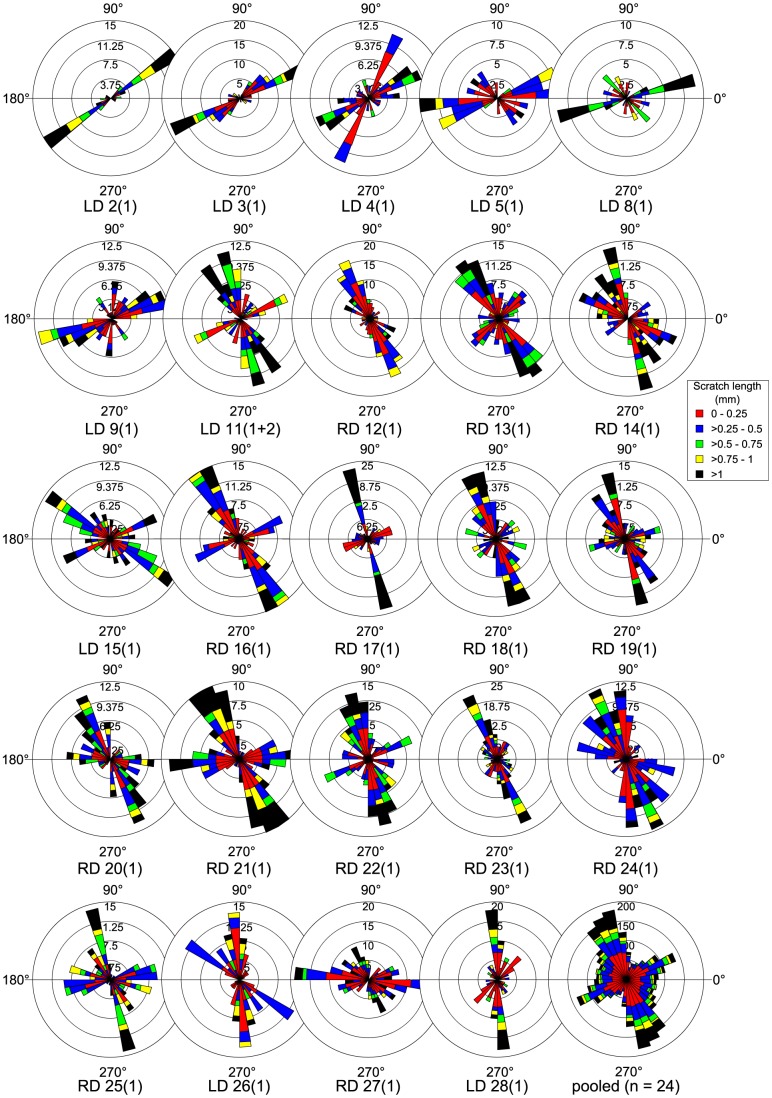
Rose diagrams depicting the orientation of microwear scratches along the tooth row of *Prosaurolophus maximus* (CMN 2870). Note that, at the position of the eleventh tooth family, the most prominent mode of scratches shifts from a dorsomesial-ventrodistal orientation mesially to a dorsodistal-ventromesial orientation distally. Where possible, scratch orientations have been standardized to correspond to teeth from the right dentary (0° =  mesial, 90° =  apical).

Given the likelihood that only those teeth in the LWZ were functional, we focused on them for further study. We did not discriminate between the positions of teeth within the LWZ (i.e., whether they are located labially or more lingually in their respective tooth families) because the occlusal surfaces of these teeth all share approximately the same inclination and therefore likely functioned in the same way [27]. The best hadrosaurid specimen available for dental microwear analysis pertains to *Prosaurolophus maximus* (CMN 2870). We recovered microwear from 24 tooth families representing most of the dentary tooth row except the distal-most extremity where the teeth remain in occlusion and are obscured by both the coronoid process and matrix. Scratch distribution is non-random and complex, but we have attempted to highlight only the salient features here (Figure 11). Individual tooth occlusal surfaces are usually characterized by uni- or bimodal scratch distributions. At positions mesial to the eleventh tooth family, the dominant mode consists of shallow scratches oriented mesiodistally (0°–40°, 180°–220°) and includes the longest scratches (>1 mm). A second mode comprising shorter, more steeply inclined (60°–140°, 240°–320°) scratches is often seen, but here scratches are much less numerous. More distally, the dominant mode consists of steep, dorsoventrally or dorsodistally-ventromesially inclined (80°–140°, 260°–320°) scratches that regularly exceed 1 mm in length. A second mode of shallow, mesiodistally oriented (0°–40°, 180°–220°; 140°–180°, 320°–360°) scratches is also regularly observed, although scratches are typically neither as numerous nor as frequently elongate. The overall pattern along the tooth row is that of a bimodal distribution (Figure 11), where the dominant mode comprises steep, dorsodistally-ventromesially inclined (100°–130°, 280°–310°) scratches, a high proportion of which are >1 mm. A second mode comprises shorter, less numerous scratches oriented mesiodistally (10°–40°, 190°–220°). The number of scratches increases slightly distally along the tooth row, from a mean of 34 in the mesial half of the tooth row to 40 in the distal half. A Pearson's correlation test suggests that the correlation of scratch count with tooth position is significant (n = 24, r = 0.603, *p*<0.05); however, this correlation becomes insignificant if teeth mesial to the fifth tooth family are excluded. A Pearson's correlation test also reveals no significant correlation between log-transformed pit count and tooth position (n = 24, r = −0.268, *p*>0.05). A Spearman's rank correlation test suggests that there is a significant correlation between tooth position and average feature width (n = 24, r = −0.381, *p*<0.05), with average feature width becoming smaller distally along the tooth row; however, the correlation becomes insignificant if the mesial-most tooth preserving microwear (LD 2) is excluded (Table S7).

**Figure 12:**
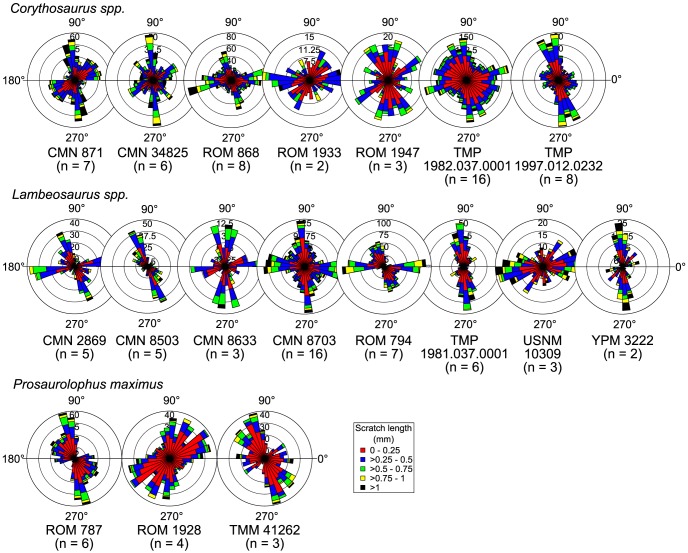
Rose diagrams depicting the orientation of pooled microwear scratches in hadrosaurids. Scratches are typically oriented dorsodistally-ventromesially; however, a second common mode comprises mesiodistally oriented scratches. Where possible, scratch orientations have been standardized to correspond to teeth from the right dentary (0° =  mesial, 90° =  apical).

Other hadrosaurid specimens possess very similar microwear patterns (Figure 12), but they do not typically exhibit the same distal shift from predominantly mesiodistally inclined to dorsodistally-ventromesially inclined scratches as in CMN 2870. Rather, long, dorsodistally-ventromesially oriented scratches tend to dominate along the length of the tooth row (*Corythosaurus casuarius*, ROM 871; *C.* sp., CMN 34825, TMP 1997.012.0232; *Lambeosaurus clavinitialis*, TMP 1981.037.0001, YPM 3222; *L.* sp., CMN 8503, CMN 8633; *Prosaurolophus maximus*, ROM 787, TMM 41262), forming the most prominent mode of scratch distribution. Mesiodistally oriented scratches are also common, and may often equal or dominate over scratches from other modes in both number and length (*C. casuarius*, ROM 1933; *C*. sp. ROM 868, TMP 1982.037.0001; *L. clavinitialis*, CMN 8703; *L. lambei*, CMN 2869, ROM 794, USNM 10309). One specimen (*P. maximus*, ROM 1928) exhibits scratches preferentially oriented dorsomesially-ventrodistally, but this signal stems from just four preserved teeth and may not necessarily reflect the pattern along the length of the tooth row. There does not appear to be any discernible systematic pattern to the scratch distribution data; rather, the same general pattern applies to all species, albeit with some intraspecific variation.

### Quantitative microwear comparisons

#### Time-averaged approach

**Figure 13:**
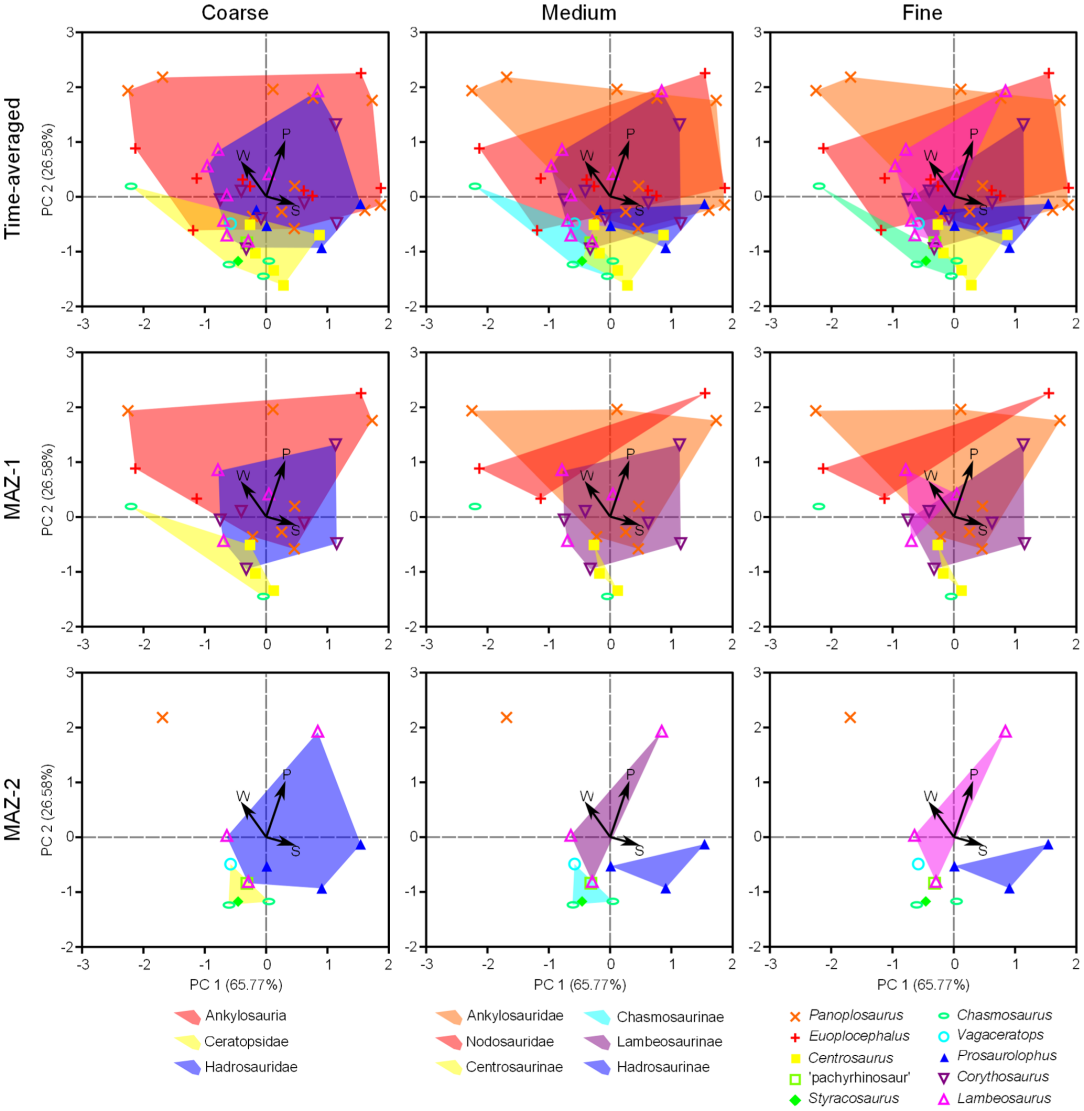
PCA biplots at coarse (suborder/family), medium (family/subfamily), and fine (genus) taxonomic scales. Arrows depict microwear feature loadings. Note that the same broad taxa occupy the same areas of microwear space through time, despite species turnover. Abbreviations: MAZ, megaherbivore assemblage zone; P, average pit count; S, average scratch count; W, average feature width.

NPMANOVA reveals significant microwear differences between ankylosaurs, ceratopsids, and hadrosaurids (N = 51, F = 3.931, *p* < 0.01). Posthoc pairwise comparisons (Table 1) indicate that ceratopsids differ significantly from ankylosaurs and hadrosaurids, but the last two taxa do not differ from one another. Further comparisons (Table 2) demonstrate that ceratopsids differ from the other taxa in having significantly fewer microwear pits (N = 51, H = 20.62, *p* < 0.0001). Interestingly, although both ankylosaurs and hadrosaurids are represented by approximately equal numbers of specimens, the former taxon occupies a considerably greater area of multivariate space than the latter, which occupies an area approximately equal in size to that of ceratopsids (Figure 13A).

**Table 1:** NPMANOVA test results for the time-averaged, suborder/family level pairwise comparisons.

	Ankylosauria	Ceratopsidae	Hadrosauridae
	(n = 20)	(n = 12)	(n = 19)
Ankylosauria (n = 20)		**0.000**	0.104
Ceratopsidae (n = 12)	**0.001**		**0.006**
Hadrosauridae (n = 19)	0.312	**0.017**	

N = 51, F = 3.931, *p* = 0.001.

Bonferroni corrected *p*-values shown in lower left triangle; uncorrected *p*-values shown in upper right triangle. Significant results reported in bold.

**Table 2:** Mann-Whitney U test results for the time-averaged, suborder/family level pairwise comparisons.

Scratches	Ankylosauria	Ceratopsidae	Hadrosauridae
	(n = 20)	(n = 12)	(n = 19)
Ankylosauria (n = 20)		0.800	0.684
Ceratopsidae (n = 12)	1		0.656
Hadrosauridae (n = 19)	1	1	
Kruskal-Wallis test: N = 51, H = 0.284, *p* = 0.867			

Pits	Ankylosauria	Ceratopsidae	Hadrosauridae
	(n = 20)	(n = 12)	(n = 19)
Ankylosauria (n = 20)		**0.000**	0.087
Ceratopsidae (n = 12)	**0.000**		**0.000**
Hadrosauridae (n = 19)	0.266	**0.001**	
Kruskal-Wallis test: N = 51, H = 20.620, *p* = **0.000**			

Widths	Ankylosauria	Ceratopsidae	Hadrosauridae
	(n = 20)	(n = 12)	(n = 19)
Ankylosauria (n = 20)		0.381	0.899
Ceratopsidae (n = 12)	1		0.530
Hadrosauridae (n = 19)	1	1	
Kruskal-Wallis test: N = 51, H = 0.780, *p* = 0.677			

Bonferroni corrected *p*-values shown in lower left triangle; uncorrected *p*-values shown in upper right triangle. Significant results reported in bold.

Increasing taxonomic resolution (Tables 3–6) also reveals interesting differences not otherwise distinguishable at coarser scales. For example, according to the uncorrected probabilities, nodosaurids possess more pits than lambeosaurines, (*p*<0.05; Table 4), but this inference must be tempered in light of the non-significant *p*-value returned by the posthoc NPMANOVA test (*p* = 0.120; Table 3). The minimal overlap of centrosaurines and chasmosaurines in multivariate space (Figure 13B) also suggests that the former may have more scratches than the latter, but statistical support is not significant (*p* = 0.051, Table 4). These shortcomings may reflect issues of small sample size. The uncorrected probabilities further indicate that the ceratopsid *Centrosaurus* differs from the hadrosaurid *Lambeosaurus* (*p*<0.001; Table 5) in having both more scratches (*p*<0.01) and finer features (p<0.05; Table 6), and that the hadrosaurine *Prosaurolophus* differs from the lambeosaurine *Lambeosaurus* (*p*<0.05; Table 5) in the same ways (*p*<0.05, Table 6).

**Table 3:** NPMANOVA test results for the time-averaged, family/subfamily level pairwise comparisons.

	Ankylosauridae	Nodosauridae	Centrosaurinae	Chasmosaurinae	Hadrosaurinae	Lambeosaurinae
	(n = 9)	(n = 11)	(n = 7)	(n = 5)	(n = 4)	(n = 15)
Ankylosauridae (n = 9)		0.799	**0.005**	0.072	0.196	0.270
Nodosauridae (n = 11)	1		**0.003**	**0.016**	0.308	0.120
Centrosaurinae (n = 7)	0.081	0.051		0.102	0.081	**0.010**
Chasmosaurinae (n = 5)	1	0.242	1		0.091	0.117
Hadrosaurinae (n = 4)	1	1	1	1		0.134
Lambeosaurinae (n = 15)	1	1	0.152	1	1	

Total analysis: N = 51, F = 2.253, *p* = 0.007.

Bonferroni corrected *p*-values shown in lower left triangle; uncorrected *p*-values shown in upper right triangle. Significant results reported in bold.

**Table 4:** Mann-Whitney U test results for the time-averaged, family/subfamily level pairwise comparisons.

Scratches	Ankylosauridae	Nodosauridae	Centrosaurinae	Chasmosaurinae	Hadrosaurinae	Lambeosaurinae
	(n = 9)	(n = 11)	(n = 7)	(n = 5)	(n = 4)	(n = 15)
Ankylosauridae (n = 9)		0.648	0.525	0.423	0.247	1
Nodosauridae (n = 11)	1		0.650	0.174	0.845	0.213
Centrosaurinae (n = 7)	1	1		0.051	0.705	**0.041**
Chasmosaurinae (n = 5)	1	1	0.770		0.111	0.485
Hadrosaurinae (n = 4)	1	1	1	1		0.064
Lambeosaurinae (n = 15)	1	1	0.614	1	0.965	
Kruskal-Wallis test: N = 51, H = 7.296, *p* = 0.199						

Pits	Ankylosauridae	Nodosauridae	Centrosaurinae	Chasmosaurinae	Hadrosaurinae	Lambeosaurinae
	(n = 9)	(n = 11)	(n = 7)	(n = 5)	(n = 4)	(n = 15)
Ankylosauridae (n = 9)		0.3021	**0.034**	**0.019**	0.757	0.655
Nodosauridae (n = 11)	1		**0.001**	**0.002**	0.212	**0.038**
Centrosaurinae (n = 7)	0.508	**0.016**		0.371	**0.047**	**0.004**
Chasmosaurinae (n = 5)	0.290	**0.033**	1		**0.020**	**0.010**
Hadrosaurinae (n = 4)	1	1	0.708	0.300		0.920
Lambeosaurinae (n = 15)	1	0.566	0.064	0.132	1	
Kruskal-Wallis test: N = 51, H = 22.18, *p* = **0.000**						

Widths	Ankylosauridae	Nodosauridae	Centrosaurinae	Chasmosaurinae	Hadrosaurinae	Lambeosaurinae
	(n = 9)	(n = 11)	(n = 7)	(n = 5)	(n = 4)	(n = 15)
Ankylosauridae (n = 9)		0.362	0.138	0.424	0.105	0.766
Nodosauridae (n = 11)	1		0.856	1	0.473	0.378
Centrosaurinae (n = 7)	1	1		0.745	0.395	0.205
Chasmosaurinae (n = 5)	1	1	1		0.391	0.601
Hadrosaurinae (n = 4)	1	1	1	1		0.064
Lambeosaurinae (n = 15)	1	1	1	1	0.965	
Kruskal-Wallis test: N = 51, H = 5.429, *p* = 0.366						

Bonferroni corrected *p*-values shown in lower left triangle; uncorrected *p*-values shown in upper right triangle. Significant results reported in bold.

**Table 5:** NPMANOVA test results for the time-averaged, genus level pairwise comparisons.

	*Euoplocephalus*	*Panoplosaurus*	*Centrosaurus*	*Chasmosaurus*	*Prosaurolophus*	*Corythosaurus*	*Lambeosaurus*
	(n = 9)	(n = 11)	(n = 5)	(n = 4)	(n = 4)	(n = 7)	(n = 8)
*Euoplocephalus* (n = 9)		0.801	**0.024**	0.125	0.193	0.340	0.443
*Panoplosaurus* (n = 11)	1		**0.025**	**0.033**	0.318	0.398	0.149
*Centrosaurus* (n = 5)	0.504	0.519		0.240	0.191	0.164	**0.001**
*Chasmosaurus* (n = 4)	1	0.689	1		0.143	0.160	0.247
*Prosaurolophus* (n = 4)	1	1	1	1		0.669	**0.037**
*Corythosaurus* (n = 7)	1	1	1	1	1		0.172
*Lambeosaurus* (n = 8)	1	1	**0.027**	1	0.777	1	

Total analysis: N = 48, F = 1.728, *p* = 0.036.

Bonferroni corrected *p*-values shown in lower left triangle; uncorrected *p*-values shown in upper right triangle. Significant results reported in bold.

**Table 6:** Mann-Whitney U test results for the time-averaged, genus level pairwise comparisons.

Scratches	*Euoplocephalus*	*Panoplosaurus*	*Centrosaurus*	*Chasmosaurus*	*Prosaurolophus*	*Corythosaurus*	*Lambeosaurus*
	(n = 3)	(n = 2)	(n = 5)	(n = 4)	(n = 4)	(n = 7)	(n = 8)
*Euoplocephalus*(n = 3)		0.773	1	0.216	0.596	0.820	0.083
*Panoplosaurus* (n = 2)	1		0.561	0.105	0.488	0.188	**0.050**
*Centrosaurus* (n = 5)	1	1		0.066	1	0.330	**0.010**
*Chasmosaurus* (n = 4)	1	1	1		0.194	0.299	0.671
*Prosaurolophus* (n = 4)	1	1	1	1		0.299	**0.033**
*Corythosaurus* (n = 7)	1	1	1	1	1		0.056
*Lambeosaurus* (n = 8)	1	1	0.219	1	0.709	1	
Kruskal-Wallis test: N = 31, H = 14.31, *p* = **0.026**							

Pits	*Euoplocephalus*	*Panoplosaurus*	*Centrosaurus*	*Chasmosaurus*	*Prosaurolophus*	*Corythosaurus*	*Lambeosaurus*
	(n = 3)	(n = 2)	(n = 5)	(n = 4)	(n = 4)	(n = 7)	(n = 8)
*Euoplocephalus* (n = 3)		0.773	**0.037**	0.052	0.216	0.172	0.126
*Panoplosaurus* (n = 2)	1		0.081	0.105	0.247	0.107	0.090
*Centrosaurus* (n = 5)	0.775	1		0.111	0.111	0.051	0.057
*Chasmosaurus* (n = 4)	1	1	1		**0.030**	**0.018**	**0.017**
*Prosaurolophus* (n = 4)	1	1	1	0.638		0.925	0.734
*Corythosaurus* (n = 7)	1	1	1	0.381	1		0.862
*Lambeosaurus* (n = 8)	1	1	1	0.362	1	1	
Kruskal-Wallis test: N = 31, H = 18.26, *p* = **0.006**							

Widths	*Euoplocephalus*	*Panoplosaurus*	*Centrosaurus*	*Chasmosaurus*	*Prosaurolophus*	*Corythosaurus*	*Lambeosaurus*
	(n = 3)	(n = 2)	(n = 5)	(n = 4)	(n = 4)	(n = 7)	(n = 8)
*Euoplocephalus* (n = 3)		0.7728	0.371	0.860	0.596	1	0.185
*Panoplosaurus* (n = 2)	1		0.333	0.817	0.488	0.884	**0.050**
*Centrosaurus* (n = 5)	1	1		0.540	0.713	0.417	**0.034**
*Chasmosaurus* (n = 4)	1	1	1		0.471	0.777	0.203
*Prosaurolophus* (n = 4)	1	1	1	1		0.395	**0.022**
*Corythosaurus* (n = 7)	1	1	1	1	1		0.056
*Lambeosaurus* (n = 8)	1	1	0.710	1	0.459	1	
Kruskal-Wallis test: N = 31, H = 10.3, *p* = 0.113							

Bonferroni corrected *p*-values shown in lower left triangle; uncorrected *p*-values shown in upper right triangle. Significant results reported in bold.

#### Time-constrained approach

Compared to the time-averaged approach, the recovered probabilities in both MAZ-1 (Tables 7–12) and -2 (Tables 13–17) are less significant, probably owing to the smaller sample size available in each assemblage zone. However, where differences are significant, they largely correspond to those of the time-averaged approach. For example, in MAZ-1, ceratopsids differ from ankylosaurs (*p*<0.05) in having fewer microwear pits (*p*<0.05). Importantly, there are no new differences recovered among taxa that might indicate that time-averaging obscures palaeoecological patterns that are otherwise discernible at fine temporal resolutions (Figure 13B, C).

**Table 7:** NPMANOVA test results for the MAZ-1, suborder/family level pairwise comparisons.

	Ankylosauria	Ceratopsidae	Hadrosauridae
	(n = 10)	(n = 5)	(n = 9)
Ankylosauria (n = 10)		**0.044**	0.452
Ceratopsidae (n = 5)	0.131		0.119
Hadrosauridae (n = 9)	1	0.356	
Total analysis: N = 24, F = 1.767, *p* = 0.115			

Bonferroni corrected *p*-values shown in lower left triangle; uncorrected *p*-values shown in upper right triangle. Significant results reported in bold.

**Table 8:** Mann-Whitney U test results for the MAZ-1, suborder/family level pairwise comparisons.

Scratches	Ankylosauria (n = 10)	Ceratopsidae (n = 5)	Hadrosauridae (n = 9)
Ankylosauria (n = 10)		0.902	0.967
Ceratopsidae (n = 5)	1		0.790
Hadrosauridae (n = 9)	1	1	
Kruskal-Wallis test: N = 24, H = 0.092, *p* = 0.955			

Pits	Ankylosauria (n = 10)	Ceratopsidae (n = 5)	Hadrosauridae (n = 9)
Ankylosauria (n = 10)		**0.006**	0.653
Ceratopsidae (n = 5)	**0.017**		**0.005**
Hadrosauridae (n = 9)	1	**0.015**	
Kruskal-Wallis test: N = 24, H = 10.14, *p* = **0.006**			

Widths	Ankylosauria (n = 10)	Ceratopsidae (n = 5)	Hadrosauridae (n = 9)
Ankylosauria (n = 10)		0.501	0.653
Ceratopsidae (n = 5)	1		1
Hadrosauridae (n = 9)	1	1	
Kruskal-Wallis test: N = 24, H = 0.524, *p* = 0.769			

Bonferroni corrected *p*-values shown in lower left triangle; uncorrected *p*-values shown in upper right triangle. Significant results reported in bold.

**Table 9:** NPMANOVA test results for the MAZ-1, family/subfamily level pairwise comparisons.

	Ankylosauridae	Nodosauridae	Centrosaurinae	Chasmosauriane	Lambeosaurinae
	(n = 3)	(n = 7)	(n = 3)	(n = 2)	(n = 9)
Ankylosauridae (n = 3)		0.612	0.101	0.803	0.147
Nodosauridae (n = 7)	1		0.130	0.279	0.732
Centrosaurinae (n = 3)	1	1		0.700	0.268
Chasmosauriane (n = 2)	1	1	1		0.351
Lambeosaurinae (n = 9)	1	1	1	1	
Total analysis: N = 24, F = 1.11, *p* = 0.358.					

Bonferroni corrected *p*-values shown in lower left triangle; uncorrected *p*-values shown in upper right triangle.

**Table 10:** Mann-Whitney U test results for the MAZ-1, family/subfamily level pairwise comparisons.

Scratches	Ankylosauridae	Nodosauridae	Centrosaurinae	Chasmosaurinae	Lambeosaurinae
	(n = 3)	(n = 7)	(n = 3)	(n = 2)	(n = 9)
Ankylosauridae (n = 3)		0.361	0.658	0.767	0.354
Nodosauridae (n = 7)	1		0.568	0.661	0.525
Centrosaurinae (n = 3)	1	1		0.773	0.355
Chasmosaurinae (n = 2)	1	1	1		0.556
Lambeosaurinae (n = 9)	1	1	1	1	
Kruskal-Wallis test: N = 24, H = 2.346, *p* = 0.672					

Pits	Ankylosauridae	Nodosauridae	Centrosaurinae	Chasmosaurinae	Lambeosaurinae
	(n = 3)	(n = 7)	(n = 3)	(n = 2)	(n = 9)
Ankylosauridae (n = 3)		0.643	0.383	0.149	0.853
Nodosauridae (n = 7)	1		**0.022**	0.055	0.458
Centrosaurinae (n = 3)	1	0.218		0.149	**0.027**
Chasmosaurinae (n = 2)	1	0.550	1		**0.045**
Lambeosaurinae (n = 9)	1	1	0.265	0.451	
Kruskal-Wallis test: N = 24, H = 10.92, *p* = **0.027**					

Widths	Ankylosauridae	Nodosauridae	Centrosaurinae	Chasmosaurinae	Lambeosaurinae
	(n = 3)	(n = 7)	(n = 3)	(n = 2)	(n = 9)
Ankylosauridae (n = 3)		0.494	0.383	0.773	0.267
Nodosauridae (n = 7)	1		0.649	0.884	1
Centrosaurinae (n = 3)	1	1		0.773	0.712
Chasmosaurinae (n = 2)	1	1	1		0.724
Lambeosaurinae (n = 9)	1	1	1	1	
Kruskal-Wallis test: N = 24, H = 1.81, *p* = 0.771					

Bonferroni corrected *p*-values shown in lower left triangle; uncorrected *p*-values shown in upper right triangle. Significant results reported in bold.

**Table 11:** NPMANOVA test results for the MAZ-1, genus level pairwise comparisons.

	*Panoplosaurus*	*Centrosaurus*	*Chasmosaurus*	*Corythosaurus*	*Lambeosaurus*
	(n = 7)	(n = 3)	(n = 2)	(n = 6)	(n = 3)
*Panoplosaurus* (n = 7)		0.396	0.331	0.528	0.098
*Centrosaurus* (n = 3)	1		0.704	0.538	0.099
*Chasmosaurus* (n = 2)	1	1		0.566	0.500
*Corythosaurus* (n = 6)	1	1	1		0.609
*Lambeosaurus* (n = 3)	0.982	0.991	1	1	
Total analysis: N = 21, F = 1.407, *p* = 0.188.					

Bonferroni corrected *p*-values shown in lower left triangle; uncorrected *p*-values shown in upper right triangle.

**Table 12:** Mann-Whitney U test results for the MAZ-1, genus level pairwise comparisons.

Scratches	*Panoplosaurus*	*Centrosaurus*	*Chasmosaurus*	*Corythosaurus*	*Lambeosaurus*
	(n = 2)	(n = 3)	(n = 2)	(n = 6)	(n = 3)
*Panoplosaurus* (n = 2)		0.387	0.245	0.243	0.149
*Centrosaurus* (n = 3)	1		0.773	0.897	0.081
*Chasmosaurus* (n = 2)	1	1		0.405	0.773
*Corythosaurus* (n = 6)	1	1	1		0.156
*Lambeosaurus* (n = 3)	1	0.809	1	1	
Kruskal-Wallis test: N = 16, H = 6.559, *p* = 0.161					

Pits	*Panoplosaurus*	*Centrosaurus*	*Chasmosaurus*	*Corythosaurus*	*Lambeosaurus*
	(n = 2)	(n = 3)	(n = 2)	(n = 6)	(n = 3)
*Panoplosaurus* (n = 2)		0.149	0.245	0.134	0.149
*Centrosaurus* (n = 3)	1		0.149	0.053	0.081
*Chasmosaurus* (n = 2)	1	1		0.067	0.149
*Corythosaurus* (n = 6)	1	0.528	0.668		0.897
*Lambeosaurus* (n = 3)	1	0.809	1	1	
Kruskal-Wallis test: N = 16, H = 11.24, *p* = **0.024**					

Widths	*Panoplosaurus*	*Centrosaurus*	*Chasmosaurus*	*Corythosaurus*	*Lambeosaurus*
	(n = 2)	(n = 3)	(n = 2)	(n = 6)	(n = 3)
*Panoplosaurus* (n = 2)		0.773	0.699	0.617	0.149
*Centrosaurus* (n = 3)	1		0.773	0.897	0.383
*Chasmosaurus* (n = 2)	1	1		0.617	0.773
*Corythosaurus* (n = 6)	1	1	1		0.245
*Lambeosaurus* (n = 3)	1	1	1	1	
Kruskal-Wallis test: N = 16, H = 2.713, *p* = 0.607					

Bonferroni corrected *p*-values shown in lower left triangle; uncorrected *p*-values shown in upper right triangle. Significant results reported in bold.

**Table 13:** Statistical test results for the MAZ-2, family level (Ceratopsidae/Hadrosauridae) pairwise comparisons.

Test	Results
NPMANOVA	N = 11, F = 1.748, *p* = 0.187
Scratches (Mann-Whitney U test)	N = 11, U = 8, *p* = 0.235
Pits (Mann-Whitney U test)	N = 11, U = 1.5, *p* = **0.017**
Widths (Mann-Whitney U test)	N = 11, U = 10, *p* = 0.411

Ankylosaurs are not included due to insufficient sample size. Significant results reported in bold.

**Table 14:** NPMANOVA test results for the MAZ-2, subfamily level pairwise comparisons.

	Centrosaurinae	Chasmosaurinae	Hadrosaurinae	Lambeosaurinae
	(n = 2)	(n = 3)	(n = 3)	(n = 3)
Centrosaurinae (n = 2)		0.592	0.800	0.691
Chasmosaurinae (n = 3)	1		0.396	0.194
Hadrosaurinae (n = 3)	1	1		0.506
Lambeosaurinae (n = 3)	1	1	1	
N = 11, F = 2.592, *p* = **0.011**				

Bonferroni corrected *p*-values shown in lower left triangle; uncorrected *p*-values shown in upper right triangle. Significant results reported in bold.

**Table 15:** Mann-Whitney U test results for the MAZ-2, subfamily level pairwise comparisons.

Scratches	Centrosaurinae	Chasmosaurinae	Hadrosaurinae	Lambeosaurinae
	(n = 2)	(n = 3)	(n = 3)	(n = 3)
Centrosaurinae (n = 2)		0.387	0.387	0.773
Chasmosaurinae (n = 3)	1		0.081	0.663
Hadrosaurinae (n = 3)	1	0.485		0.190
Lambeosaurinae (n = 3)	1	1	1	
Kruskal-Wallis test: N = 11, H = 5.288, *p* = 0.152				

Pits	Centrosaurinae	Chasmosaurinae	Hadrosaurinae	Lambeosaurinae
	(n = 2)	(n = 3)	(n = 3)	(n = 3)
Centrosaurinae (n = 2)		0.773	0.149	0.149
Chasmosaurinae (n = 3)	1		0.081	0.268
Hadrosaurinae (n = 3)	0.894	0.485		0.825
Lambeosaurinae (n = 3)	0.894	1	1	
Kruskal-Wallis test: N = 11, H = 6.455, *p* = 0.089				

Widths	Centrosaurinae	Chasmosaurinae	Hadrosaurinae	Lambeosaurinae
	(n = 2)	(n = 3)	(n = 3)	(n = 3)
Centrosaurinae (n = 2)		0.387	0.149	0.773
Chasmosaurinae (n = 3)	1		0.190	0.383
Hadrosaurinae (n = 3)	0.894	1		0.081
Lambeosaurinae (n = 3)	1	1	0.485	
Kruskal-Wallis test: N = 11, H = 6.424, *p* = 0.093				

Bonferroni corrected *p*-values shown in lower left triangle; uncorrected *p*-values shown in upper right triangle.

**Table 16:** NPMANOVA test results for the MAZ-2, genus level pairwise comparisons.

	*Chasmosaurus*	*Prosaurolophus*	*Lambeosaurus*
	(n = 3)	(n = 3)	(n = 3)
*Chasmosaurus* (n = 3)		0.494	0.401
*Prosaurolophus* (n = 3)	1		0.501
*Lambeosaurus* (n = 3)	1	1	
Total analysis: N = 9, F = 2.202, *p* = **0.035**			

Bonferroni corrected p-values shown in lower left triangle; uncorrected *p*-values shown in upper right triangle. Significant results reported in bold.

**Table 17:** Mann-Whitney U test results for the MAZ-2, genus level pairwise comparisons.

Scratches	*Chasmosaurus*	*Prosaurolophus*	*Lambeosaurus*
	(n = 2)	(n = 3)	(n = 3)
*Chasmosaurus* (n = 2)		0.149	0.773
*Prosaurolophus* (n = 3)	0.447		0.190
*Lambeosaurus* (n = 3)	1	0.571	
Kruskal-Wallis test: N = 8, H = 3.778, *p* = 0.151			

Pits	*Chasmosaurus*	*Prosaurolophus*	*Lambeosaurus*
	(n = 2)	(n = 3)	(n = 3)
*Chasmosaurus* (n = 2)		0.149	0.236
*Prosaurolophus* (n = 3)	0.447		0.825
*Lambeosaurus* (n = 3)	0.708	1	
Kruskal-Wallis test: N = 8, H = 3.535, *p* = 0.164			

Widths	*Chasmosaurus*	*Prosaurolophus*	*Lambeosaurus*
	(n = 2)	(n = 3)	(n = 3)
*Chasmosaurus* (n = 2)		0.387	0.149
*Prosaurolophus* (n = 3)	1		0.081
*Lambeosaurus* (n = 3)	0.447	0.243	
Kruskal-Wallis test: N = 8, H = 5.556, *p* = 0.062			

Bonferroni corrected *p*-values shown in lower left triangle; uncorrected *p*-values shown in upper right triangle.

## Discussion

There is some apprehension in the literature about the use of the word mastication (or chewing) as it applies to non-mammals (e.g., [77]–[79]). As traditionally used, mastication refers to the unilateral breakdown of food using transverse motions of the mandible and the implementation of tribosphenic molars [80], [81]. Because mammals are the only clade to possess such a system, they are essentially the only ‘true’ masticators. However, if the purpose of mastication is to both reduce food to a condition suitable for swallowing and to increase surface area for digestive enzymes [82], then mastication, in the functional sense, is common to many amniotes besides mammals [83]. For this reason, and to facilitate discussion, we do not resist using the term here in reference to dinosaurs.

### Feeding in *Ankylosauria*

#### Jaw mechanics

Due to their simple, phylliform teeth, akinetic skulls, and unspecialized jaw musculature [84], ankylosaurs are traditionally thought to have possessed correspondingly simple jaw mechanics. They have been variously described as “puncture-crushers” [85] or “orthal pulpers” [86], whereby the mandible pivots about the jaw joint in an arcilineal or orthal fashion to bring the teeth into occlusion [83]. Barrett [65] cited the presence of paired dental wear facets and apicobasally oriented microwear scratches as evidence for simple, interlocking tooth occlusion, but others [58], [87], [88] have questioned whether the teeth occluded precisely at all. This system has been compared to that of iguanids [89], [90]; however, despite the superficial similarities between ankylosaur and iguanid teeth, it is evident from tooth wear patterns that they were employed in very different ways. Iguanid tooth wear is extremely rare, if not non-existent ([91]; JCM, pers. obs.), but ankylosaur teeth regularly show signs of extensive wear, and occasionally crowns have worn down to the cingulum.

Rybczynski and Vickaryous [66] challenged the traditional view of ankylosaur jaw mechanics with reference to a well-preserved skull of the ankylosaurid *Euoplocephalus tutus* (AMNH 5405). They suggested that the power stroke in this species was palinal (retractive), citing three lines of tooth wear evidence in favour of their interpretation: (1) the presence of continuous wear facets between adjacent teeth; (2) the occurrence of a stepped enamel-dentine interface on the distal edge of maxillary teeth; (3) the presence of low-angled, mesiodistally oriented microwear scratches on dental wear facets. This jaw mechanism was thought to have been facilitated via translational movements about the jaw joint, as the mandible slid along its articulation with the quadrate. To overcome the medial bowing of the tooth rows, some medial displacement of the mandibular rami would also have been necessary [66].

The evidence presented here generally supports the findings of Rybczynski and Vickaryous [66], with two caveats. First, we were unable to verify the existence of a stepped enamel-dentine interface, which is the only cited evidence that requires a palinal (as opposed to proal) power stroke. This might be attributable to the fact that we were working at a lower magnification than Rybczynski and Vickaryous [66]. Second, although the dental wear facets of AMNH 5405 appear continuous across teeth, it must be borne in mind that, because the teeth are not tightly appressed as in ceratopsids or hadrosaurids, it is difficult to determine with certainty whether the wear facets are truly continuous between teeth or if their coplanarity is owed to the fact that the tooth rows were partially realigned so that the teeth met in almost perfect opposition, as suggested for the early thyreophoran *Scelidosaurus harrisonii* [65]. Individual wear features do not appear continuous across teeth, as in ceratopsids or hadrosaurids.

Nodosaurid teeth do not typically possess the same vertical, planar wear facets as ankylosaurids. Rather, the facets tend to cut obliquely across the mesial or distal edge of the tooth. Nevertheless, the dental microwear evidence suggests that ankylosaurids and nodosaurids shared similar jaw mechanics, which were more complex than traditionally assumed. Certainly, some orthal or arcilineal motion of the mandible was possible, as revealed by the existence of dorsoventrally or dorsodistally-ventromesially inclined scratches. These scratches align with the resultant vector of the external adductor musculature of the mandible [14], [84], which would have acted to bring the teeth into occlusion. However, the ubiquitous presence of low angled, mesiodistally inclined scratches strongly supports the interpretation of Rybczynski and Vickaryous [66] that some form of propalinal motion was also possible. This motion would have been effected by the complementary action of the pterygoideus musculature, which pulled the mandible rostrally, and the posterior adductor musculature, which pulled the mandible caudally. Occasional dorsomesially-ventrodistally inclined scratches likewise align with the resultant vector of the pterygoideus musculature, and may have formed during repositioning movements of the masticatory cycle [92]. Those differences in macrowear between ankylosaurids and nodosaurids might simply reflect differences in tooth alignment, rather than jaw mechanics.

#### Diet

Ankylosaurs are one of several clades of dinosaurs whose feeding behaviour has been interpreted within the ‘iguanine paradigm’ [93], a reflection of their phylliform teeth. As such, ankylosaurs are traditionally thought to have fed on soft, succulent plant tissues similar to those consumed by iguanines [63], [84], [89], [90]. However, differences in the tooth morphology of ankylosaurids and nodosaurids suggest that ankylosaur diets were more varied than previously assumed [94], the specifics of which have not yet been examined. The following is a preliminary attempt to explore the functional differences between ankylosaurid and nodosaurid teeth, and to gain some insight into their palaeoecological ramifications.

Ankylosaur teeth vary in both size and shape; whereas unworn ankylosaurid teeth tend to be small and pointed or cusp-like, those of nodosaurids tend to be larger and more blade-like. Lucas ([17]:p. 169) noted that tooth size probably most closely reflects food particle size, especially as it relates to the concept of “breakage sites”. The argument is as follows: because small particles are less likely to be fractured in the jaws than larger ones, the most obvious evolutionary response to the mastication of small particles is to develop larger teeth, thereby providing increased surface area for fracture (breakage sites). Therefore, animals with large teeth are expected to consume relatively small particles, whereas those with small teeth are expected to consume relatively large particles. Plant tissues that might be considered small include leaves and stems because they are particularly thin and possess tiny volumes, whereas large plant tissues encompass fruits and seeds [17]. From these admittedly simplistic mechanical considerations, it might be surmised that ankylosaurids incorporated more fruit in their diet, whereas nodosaurids consumed more leaves.

These dietary assignments appear to hold up in light of their associated tooth shapes. For example, the small, pointed or cusp-like morphology of ankylosaurid teeth is in some ways reminiscent of mammalian frugivore molars [95]–[98]. Fruit flesh is relatively fragile because it contains less fibre (cell wall content) compared to plant structural tissues [17], meaning that cracks propagate relatively easily through it [16]. Given this, the simplest design for fracturing fruit flesh is that of a cusp, which concentrates forces over the surface of the tissue to initiate fracture [17]. By contrast, the more blade-like qualities of nodosaurid teeth may be considered an evolutionary response to a tougher, more fibrous diet. Unlike fruit flesh, plant structural tissues like stems and leaf veins are difficult to fracture because their thinness and increased fibre content tend to inhibit crack growth (see discussion below with respect to ceratopsids). It therefore seems likely that nodosaurids incorporated more foliage in their diets than ankylosaurids. This interpretation is further buoyed by the fact that nodosaurid teeth possess an enlarged basal cingulum, which tends to occur among frequent consumers of tough, pliant foliage, and is thought to aid in the prevention of abfraction fractures near the gingiva [99].

In spite of the foregoing discussion, the microwear evidence suggests that ankylosaur families did not differ in their dietary requirements. For instance, if ankylosaurids regularly consumed more fruit than nodosaurids, they might be expected to exhibit signs of increased microwear pitting, much like frugivores do today [46], [48]–[50]. Although sample size remains small, microwear comparisons nevertheless yield no convincing support for the contention that ankylosaurids and nodosaurids specialized on different plant tissues. The regular occurrence of pitting in all ankylosaur teeth suggests that these animals ate fruit at least occasionally, in addition to more abundant foliage.

Another aspect of ankylosaur microwear worth considering is why this taxon occupies a greater area of microwear space than the similarly represented hadrosaurids (Figure 13A). Two explanations are possible. First, ankylosaur diets may have been more varied than those of their megaherbivorous counterparts, perhaps varying seasonally in response to shifting food resources. Second, ankylosaur microwear disparity might simply reflect corresponding variation in wear facet development, as seen along the tooth row of *Panoplosaurus* (see Results above). In light of the fact that ankylosaur teeth do not appear to be suitably adapted to processing the toughest woody browse attributed to ceratopsids and hadrosaurids below, the latter scenario is probably more parsimonious.

One final issue bears mentioning with respect to ankylosaur feeding. Carpenter [100] reported on the existence of gastroliths in a specimen of *Panoplosaurus mirus* (ROM 1215), but these features are not mentioned in either the field notes or resulting description [63]. Hence, there is some doubt concerning the association of the gastroliths (K. Seymour, pers. comm., 2011). Regardless, assuming the gastroliths are truly associated with the specimen, they yield important consequences for the inference of form-function relationships in the teeth of these animals. Specifically, the existence of gastroliths in ankylosaurs implies that these animals comminuted plant matter in the gizzard, and probably did not masticate their food completely. This, in turn, suggests that ankylosaurs may have ingested more resistant plant matter than their teeth convey, causing some uncertainty for the functional inferences made here.

### *Feeding in* *Ceratopsidae*

#### Jaw mechanics

It is evident from the continuous vertical occlusal surfaces of ceratopsid dental batteries that their teeth did not act individually; rather, they functioned collectively as a unit, acting as complementary blades to produce a strict shearing action, reminiscent of a pair of scissors. For this reason, many authors have envisioned ceratopsid jaws as having acted in a similar fashion, with the mandible rotating in an arcilineal fashion about the jaw joint to bring the teeth into occlusion [68], [70], [101]–[105]. However, the scissor analogy must not be taken too far because the jaw joint is offset ventrally from the tooth row, which would have constrained the teeth to occlude in a nearly parallel fashion [42]. A similar situation exists among ungulates, in which the jaw joint is offset dorsally from the tooth row, but there is little consensus concerning its adaptive significance. It may have improved the leverage of the jaw adductors [106], although some have also suggested that it allowed for the sub-equal distribution of bite forces along the jaw [107], [108], for the same set of bilaterally symmetrical muscles to move the mandible in two different directions [109], or for the accommodated increase in muscle length as a function of increased body size in accordance with fracture scaling theory [17].

In spite of the orthal jaw mechanism traditionally attributed to ceratopsids, some authors [110]–[113] have noted the existence of dental microwear with a mesiodistal component to the scratches, suggestive of some degree of propalinal motion; however, these observations were never quantified. Recently, Varriale [42] used discriminant function analysis to demonstrate the existence of polymodal scratch distributions on ceratopsid teeth, confirming previous suspicions that the orthal model of ceratopsid jaw mechanics is overly simplistic. He found evidence for the existence of four discrete modes of microwear scratches, which he termed “classes”. Correcting for the standard used here, his classes were as follows: Class 1 (range  = 89.6°–121°, mean  = 109.3°); Class 2 (range  = 121°–180°, mean  = 139.3°); Class 3 (range  = 180°–232.4°, mean  = 214.8°); Class 4 (range  = 232.4°–269.4°, mean  = 256.2°).

In the present study, most scratches are oriented dorsodistally-ventromesially, and fall within the range of Varriale's Class 1. The longest features also tend to be inclined in this direction. Therefore, it is likely that these scratches were made during the power stroke, which occurred in an orthopalinal fashion. This interpretation is in line with that of Varriale [42], who also noticed that Class 1 scratches tend to be wider and more parallel than those in other classes. Not surprisingly, Class 1 scratches align with the resultant vector of the external jaw adductor musculature [14], [102], which would have acted to bring the teeth into occlusion.

The least common scratch mode is oriented dorsomesially-ventrodistally, corresponding to Varriale's Class 4, and although this mode may occasionally include the longest features, their rarity suggests that they were not formed as a result of the power stroke. Varriale ([42]:p. 317) proposed that Class 4 scratches formed during jaw depression, referring to them as “disengagement scratches”. It is also possible that they were formed as food was occasionally repositioned within the jaws, between power stroke cycles [92]. Such movements probably would have been effected in part by the pterygoideus musculature, which pulls dorsorostrally [14], [102].

The mesiodistally inclined scratches commonly observed in this study fall within the range of Varriale's Class 2 and 3 scratches. Varriale [42] noted that his Class 2 scratches, like those of Class 1, tend to be numerous, long, wide, parallel, and are more often in line with the external adductor musculature vector resultant than not. This led him to believe that Class 2 scratches were formed during the power stroke. Conversely, his Class 3 scratches are fewer, shorter, thinner, and more variably oriented, like those of Class 4, leading him to suggest that they were formed during mandibular depression. It must be noted, however, that the Class 2 and 3 scratches of the present study more closely resemble each other than one of the other two classes. Here, Class 2 and 3 scratches adhere more closely to the mesiodistal axis, and can be quite long (>1 mm). In fact, they may occasionally be longer and outnumber Class 1 scratches (e.g., CMN 344 in Figure 8). This indicates that these scratches were probably formed during propalinal excursions of the mandible, and were perhaps even associated with the power stroke.

There are two possible mechanisms that may account for the existence of mesiodistally oriented scratches on ceratopsid teeth. The first is a passive mechanism whereby the mandible is pushed palinally as the predentary traces a dorsocaudal arc defined by its contact with the inner surface of the rostral bone during adduction [67], [111]. However, this mechanism predicts the existence of similarly curved scratches that are almost never seen, thereby rendering this explanation unlikely. A more likely explanation involves occasional propalinal movements of the adducted mandible, effected by the complementary actions of the pterygoideus and posterior adductor musculature [83], [102]. However, because ceratopsid tooth rows diverge distally, palinal movements of the mandible would have disengaged the teeth. Varriale [113] therefore suggested that any palinal movements of the mandible during the masticatory cycle must have occurred anisognathously, whereby only those teeth on one side of the dentition were in occlusion at any one time (unilateral mastication). Although anisognathy is common among mammals [79], it is not observed among living archosaurs, and is therefore highly unparsimonious to infer within Ceratopsidae [42], [114], [115]. Regardless, such movements appear to be required to account for the presence of mesiodistally oriented scratches, and would not have required displacements of more than a few millimetres at the jaw joint, which could easily have been accommodated ([42]:fig. 5.14). Therefore, ceratopsid mastication comprised an orthopalinal power stroke, possibly supplemented by occasional propalinal excursions of the mandible. This pattern appears to characterize all ceratopsids, given the lack of systematic differences among them.

#### Diet

The ceratopsid dentition is unique in that it produced a strict shearing action; crushing or grinding functions were precluded [42], [68], [70], [101], [110]. Ostrom ([102]:p. 6) noted that shearing dentitions are typical of carnivorous mammals (e.g., carnassial teeth of carnivorans and creodonts), but that “shear is of only minor or secondary importance in most herbivorous species”, which typically possess grinding or crushing dentitions. Nevertheless, Ostrom [70], [102] maintained the traditional view that ceratopsids were herbivorous, and that they must have subsisted on particularly tough and resistant plant matter.

From a mechanical standpoint, blades are suitable for fracturing foodstuffs high in toughness, which is defined as the resistance to crack propagation [16], [17]. The toughness of animal skin is largely due to its high Poisson's ratio, a measure of the narrowing or bulging of a material at right angles to stress [16]. Thus, skin stretches when pulled and bulges when compressed. This behaviour tends to suppress crack growth so that cracks do not self-propagate through skin under compression. Therefore, sharp blades are required to overcome this toughening mechanism by continually forcing crack growth through the skin. Long or inflexed blades also help to overcome the toughening mechanism imparted by a high Poisson's ratio, which explains the size and shape of mammalian carnassial teeth [17], [116].

There are several reasons to suspect that ceratopsids were not adapted to carnivory in spite of their shearing dentitions. First, these dinosaurs likely held their forelimbs in a semi-sprawled posture [103], [117]–[120] and probably could not have attained sufficient speeds for capturing animal prey [121] (but see Paul and Christiansen [122]). Second, ceratopsids possessed other features thought to be related to herbivory, such as an edentulous beak, tall coronoid process, and the aforementioned jaw joint offset from the tooth row. Third, there is evidence by way of tooth morphology and gastroliths that more basal ceratopsians were probably herbivorous [123], suggesting that herbivory was plesiomorphic for Ceratopsidae. Finally, it seems that ceratopsids were particularly abundant in their respective palaeoecosystems [124]–[126], and probably lived in massive herds at least occasionally [127]–[130], which are attributes uncharacteristic of carnivores. Therefore, if ceratopsids truly were herbivorous, the question arises as to what plant tissues their specialized bladed dentition was adapted.

Plant tissues have low Poisson's ratios [17], and therefore do not suppress crack growth in this manner. Instead, the toughness of plant tissue is a consequence of the composite cell wall and tissue structure [131], which tends to buckle in advance of growing crack tips, thereby dissipating strain energy and suppressing crack propagation (this mechanism is most effective in woody tissues). Particularly thin plant tissues (<0.5 mm), such as leaves and twigs, also exhibit elevated toughness because their thinness renders them incapable of storing enough strain energy to make cracks self-propagate, and therefore require blades for fracture [17], [132]. This probably explains why bladed or lophed teeth are common in modern folivores [19], [95], [96], [133]–[137]. Therefore, it is likely that ceratopsids also specialized in consuming tough, woody browse, including abundant leaf material. Their elevated tooth carinae would have served to trap plant tissues between them in advance of the shearing power stroke [42], [138]. This interpretation of ceratopsids as specialized consumers of particularly fibrous vegetation agrees with that of most authors [42], [68], [70], [85], [102]–[104], [111], [139], [140]–[145], although the role of fracture mechanics in explaining tooth shape has so far been underappreciated. The suggestion that ceratopsids regularly fed on more succulent vegetation [89], [141], [146]–[148]—particularly fruits—is not supported here.

The microwear evidence also supports the interpretation of ceratopsids as tough browse specialists. Folivorous mammals typically retain fewer pits on their teeth relative to those forms that include fruit in their diet [46], [48]–[50]. Ceratopsids likewise possess fewer pits than their megaherbivorous counterparts (Figure 13), suggestive of a folivorous lifestyle. The greater number of microwear scratches in centrosaurines than in chasmosaurines, although not significant, suggests that the former taxon may have subsisted on a more abrasive diet than the latter. Alternatively, it is possible that centrosaurines chewed their food more thoroughly than chasmosaurines, resulting in a higher incidence of scratches. Currently, these two competing hypotheses are underdetermined [149] by the available data. Walker [150] also noted that terrestrial primates tend to exhibit more heavily scratched teeth than arboreal forms because of the greater accumulation of exogenous grit at ground level. Feeding height stratification does not appear to be a good discriminator of centrosaurines and chasmosaurines, however, because both taxa were restricted to feeding no higher than ∼1 m above the ground [11].

The inference of ceratopsid diets from microwear nevertheless must be tempered in light of the function of their teeth. Given the strict shearing action of the ceratopsid dentition, scratches are expected to develop even if these animals were eating fruits or seeds, because the creation of pits typically requires some crushing component that the ceratopsid dentition did not possess [17]. Therefore, ceratopsid microwear is expected to more strongly reflect a functional rather than dietary signal. Conversely, because a bladed dentition is best suited to the processing of woody browse [17], such a diet is plausibly attributed to these animals.

With these considerations in mind, it is actually somewhat surprising that ceratopsid teeth are scored by pits at all. Perhaps those few pits that are present resulted from lateral movements of the mandible during anisognathus occlusion, where food particles became compressed between the maxillary and dentary tooth rows. Alternatively, it is possible that ceratopsid tooth occlusion was not as precise as is typically assumed, which may have impeded the shearing function of the teeth.

### *Feeding in* *Hadrosaurids*

#### Jaw mechanics

The subject of hadrosaurid jaw mechanics has received much attention, likely due to the interest engendered by the uniquely complex dental batteries of these animals. Ostrom [73] and Weishampel [26], [151] reviewed early models of hadrosaurid jaw mechanics [74], [152]–[157] that have since fallen out of favour for various reasons discussed therein. Currently, two models of hadrosaurid jaw mechanics have found acceptance in the literature. The first is that of Ostrom [73], which describes a power stroke employing a primarily propalinal motion of the mandible. Ostrom cited three lines of evidence in favour of his interpretation: (1) the difference in length (up to 15 mm) between opposing dental occlusal surfaces; (2) the presence of short longitudinal (mesiodistal) scratches on dental occlusal surfaces; (3) the existence of a longitudinal groove on the occlusal surface of the mandibular dentition. Ostrom envisioned all translational motion as having occurred at the jaw joint alone; the remainder of the skull was described as akinetic, owing to perceived complex and extensive contacts between the individual cranial bones. Ostrom's description of the hadrosaurid skull as akinetic has recently found favour with several workers [158], [159], and some (e.g., [75]) prefer his propalinal model of jaw mechanics.

The second model to gain broad acceptance is that of Weishampel [26], [151], who advocated a jaw mechanism apparently common among ornithopods, called pleurokinesis [160], in which tooth occlusion occurred in conjunction with lateral flaring of the maxillae and streptostylic (laterocaudal) motion of the quadrates. In addition to the consideration of joint morphology, Weishampel cited four lines of tooth wear evidence in favour of his interpretation: (1) the labial and lingual placement of enamel on maxillary and dentary teeth, respectively; (2) a flush enamel-dentine interface; (3) concave wear surface of the dentary teeth; (4) labiolingually oriented scratches on the occlusal surfaces of the teeth. The pleurokinetic model has since become widely cited in the literature (e.g., [27], [72], [79], [83], [161]).

Although both Ostrom [73] and Weishampel [26], [151] cited tooth wear evidence in favour of their models, Williams et al. [27] were the first to study hadrosaurid microwear quantitatively. With reference to several isolated jaws attributed to *Edmontosaurus*, the authors investigated how microwear signals vary within and between teeth. They found that only scratches were present, the orientations of which fell into four discrete, non-overlapping classes that they distinguished using discriminant function analysis. Adjusting to the standard used here, the classes were as follows: Class 1 (mean  = 155.86°); Class 2 (mean  = 116.71°), Class 3 (mean  = 62.70°); Class 4 (mean  = 15.43°). Thus, the apparently conflicting observations of Ostrom [73] and Weishampel [26], [151] concerning the directionality of hadrosaurid microwear can be attributed to the multimodality of the scratches. Williams at al. [27] reasoned that the steep, dorsodistally-ventromesially oriented Class 2 scratches were formed during the power stroke because these features were the most numerous, coarsest, and exhibited a high degree of parallelism. By contrast, Class 3 scratches, being lower in all these categories, were interpreted as having formed during jaw opening. Finally, Class 1 and 4 scratches were thought to have formed during slight propalinal excursions of the mandible. The authors suggested that their microwear data best fit the pleurokinetic model of Weishampel [26], [151], given the sub-dominant role that propaliny evidently played during mastication. However, it must be noted that the observations that Williams et al. [27] cited in favour of the pleurokinetic model are also in complete agreement with a simpler orthopalinal model of mastication. The authors questioned whether the mandibular rami were capable of rotating about their long axes, as has sometimes been proposed in the literature [75], [154], [155], [157], due to the lack of curved scratches. On the other hand, curved scratches would also be expected of the pleurokinetic model, given that the maxillae are said to rotate about their long axes.

The results of the current study generally support the findings of Williams et al. [27], albeit with some important exceptions. When microwear data are pooled for a single specimen, bimodal—rather than polymodal—scratch distributions are usually recovered (Figures 11–12). The predominance of dorsoventrally to dorsodistally-ventromesially inclined scratches, combined with their great lengths and persistence across nearly all hadrosaurid specimens, strongly supports the contention of Williams et al. [27] that this was the primary direction of the power stroke. Evidently, the mandible moved in an orthopalinal fashion during tooth occlusion, facilitated in part by the depressed jaw joint relative to the occlusal surface of the teeth [107]. Given that these scratches roughly parallel the vector resultant of the external adductor musculature [14], [73], [158], [162], it is likely that these muscles were recruited in the execution of the power stroke.

The common presence of mesiodistally oriented scratches also strongly suggests that the mandible was capable of supplemental propalinal motion [27]. In fact, it appears that this motion was preferentially directed palinally, as evidenced by the stepped enamel-dentine interface on the mesiolabial occlusal tooth surfaces of some specimens (e.g., *Corythosaurus casuarius*, ROM 1933; *C. intermedius*, TMP 1982.037.0001, 1992.036.0250; *C.* sp., ROM 1947; *Lambeosaurus clavinitialis*, CMN 8703). Weishampel [26] rejected hadrosaurid propalinaly, arguing that such motion would require a mesiodistal disposition of enamel on the occlusal surfaces of the teeth, providing a more effective triturating surface. Regardless, it is evident that these animals did regularly employ propaliny during mastication in spite of the lack of mesiodistally placed enamel on their teeth. Numerous explanations might be offered to account for the lack of this adaptation, including functional or developmental constraints [163].

Further evidence in favour of propaliny, originally noted by Ostrom [73], is the presence of a “longitudinal groove” along the length of the dentary tooth row ([26]:p. 62): an analogous situation exists in the ceratopsian *Leptoceratops gracilis*, which possesses a horizontal shelf on the dentary teeth that was recently shown to have developed partly as a result of propalinal jaw movements [42]. Importantly, it is also possible that such a feature may develop in the absence of propaliny if occluding teeth simply do not shear completely past one another, creating a differential wear pattern along the length of the tooth row. Even so, the other tooth wear data strongly indicate that the longitudinal groove of the hadrosaurid mandibular dentition resulted from propaliny. Given the typical reptilian nature of hadrosaurid adductor musculature [14], [73], [162], propalinal mandibular movements were likely effected by the complementary action of the posterior mandibular adductor and pterygoideus musculature [73].

Finally, the distal shift from dorsomesially-ventrodistally to dorsodistally-ventromesially oriented microwear scratches at the position of the eleventh tooth family in CMN 2870 (*Prosaurolophus maximus*) requires explanation. It is difficult to imagine how this pattern might emerge during mastication because the dentary was akinetic so that teeth at either end of the tooth row would have moved in the same direction. Instead, we suggest that the dorsomesially-ventrodistally inclined scratches at the mesial end of the tooth row were formed during browsing. If a branch was gripped between the mesialmost teeth and the head was flexed to strip leaves from the bark, this might cause the branch to scrape across the teeth as the head pulled away, leaving microwear scratches with the observed inclination.

#### Diet

Among other things, hadrosaurid dental batteries differ from those of ceratopsids in their possession of obliquely inclined wear facets, suggestive of the potential for crushing functions in addition to shearing. Assuming a wear facet that is angled 50° from the horizontal (a reasonable value for most hadrosaurids [26], [72]), basic vector decomposition shows that, for a given force applied normal to the occlusal surface, 54% would be allotted to shearing, whereas 46% would be allotted to crushing. These values are similar to those reported for mammalian folivores/frugivores [19], and it is likely that hadrosaurids may have possessed a comparable diet. The tessellated occlusal surface of the teeth, in combination with occasional propalinal movements of the mandible, would have enhanced the shredding of fibrous plant materials. The interpretation of Morris [164] that hadrosaurids were adapted to feeding on mollusks and small crustaceans, in addition to plant tissues, is not supported here because such durophagous habits typically require a predominantly crushing dentition [17], [165].

The characterization of hadrosaurids as folivores/frugivores generally agrees with the microwear data presented here. Relative to ceratopsids, which are interpreted as strict high-fibre browsers (see Feeding in Ceratopsidae above), hadrosaurids possess a higher incidence of pits on their teeth. (Incidentally, Williams et al. [27] reported an absence of microwear pitting in their hadrosaurid sample, whereas Fiorillo [41] reported a slightly higher incidence of pitting than what we have recovered here. The discrepancies might be attributable to methodological differences in working magnification.) Although the relationship between the physical properties of foods and dental microwear is not yet fully understood, evidence from the primate microwear literature [48], [50] suggests that pitting typically results from the ingestion of “hard” foodstuffs, such as seeds, even if only indirectly via the consumption of the fruits that they encapsulate. Therefore, it seems likely that hadrosaurids possessed a more generalized diet than ceratopsids, consuming fruits and seeds in addition to leaves and stems. Hadrosaurid diets may have been more like those of ankylosaurids, as implied by the fact that the two clades overlap considerably in microwear space. The distinction between ceratopsid and hadrosaurid microwear suggests that the convergent evolution of dental batteries in these clades may not necessarily have been in response to the consumption of similar plant fodder [143].

The interpretation of hadrosaurids as generalist folivores/frugivores accords with known examples of putative hadrosaurid gut contents, which contain conifer and angiosperm twigs and stems, bark, seeds, and leaves [166]–[168]. However, given concerns regarding the possible taphonomic origin of some of these materials [167], [169], the gut contents must be interpreted cautiously. Chin and Gill [170] and Chin [171] also reported on hadrosaurid coprolites containing an abundance of conifer wood, which cannot have been derived allochthonously. The larger number of microwear scratches, and their finer quality, in *Prosaurolophus* intimates that they may have possessed a less coarse, but grittier, diet than *Lambeosaurus*. Alternatively, it may reflect the fact that *Prosaurolophus* habitually fed at lower heights than *Lambeosaurus*, where small exogenous grit particles are most abundant [150],[172]. If so, this could corroborate the finding of Carrano et al. [173] that hadrosaurines typically occupied open habitats. Sample size remains small (N = 19), and more data are necessary to decide between these two hypotheses.

### A note on dinosaur microwear

Williams et al. [27] noted with reference to an in situ hadrosaurid dentition that the microwear signal in an area of 0.1 mm^2^ provides a reasonably representative sample of both the individual tooth and entire tooth row. For this reason, they justifiably concluded that microwear studies could be conducted on isolated teeth. However, the results presented here suggest that this optimism is not entirely warranted. The microwear signal in one area of a tooth often differed from that in another area of the same tooth; likewise, the signal from a tooth in one part of the tooth row often differed from that in another part of the tooth row, irrespective of the taxon considered. Therefore, while it is possible that an isolated tooth may preserve a microwear signal representative of the entire tooth row, this is evidently not always the case. When conducting dental microwear analysis on dinosaurs or other homodont taxa, we recommend examining several teeth from the dentition (if possible) to account for potential variation along the tooth row.

### Evolution of propaliny in dinosaurs

Several classic reviews of herbivorous dinosaur jaw mechanics [26], [79], [85], [89], [142], [174] have emphasized the importance of the evolution of different jaw mechanisms in accounting for the success of various groups (e.g., lower jaw rotation in *Heterodontosaurus*, pleurokinesis in *Hypsilophodon* and hadrosauroids, propaliny in psittacosaurids, etc.). However, comparatively little attention has been given to the common mechanisms that undergird the success of these various groups, likely because they had not been previously recognized. The inferred presence of propaliny in the ankylosaurs, ceratopsids, and hadrosaurids studied here strongly attests to the likelihood that such fore-aft movements of the lower jaw were common to all Genasauria (sensu Sereno [175]), having been inherited from a common ancestor. Indeed, some degree of propaliny has been noted in certain saurischians as well (e.g., [176]–[178]), which may indicate that this jaw mechanism was primitive for all dinosaurs. Further study of basal ornithischian and saurischian jaw mechanics will help to clarify the primitive state for Dinosauria, and may provide some explanatory power for their evolutionary success.

### Evolutionary palaeoecology

The degree to which the megaherbivorous dinosaurs from the DPF may have differed in their dietary requirements depends on whether tooth morphology or wear is considered. For example, in considering only unworn tooth morphology, four morphotypes can be discerned that might reasonably be expected to reflect different dietary preferences. The ankylosaurid and nodosaurid ankylosaurs each possess unique tooth morphologies—differing mainly in such features as size, bladedness, and degree of denticulation—that are otherwise known to correlate with the internal mechanical properties and external physical attributes of food [17]. Ceratopsid and hadrosaurid teeth likewise differ from those of ankylosaurs in forming complex dental batteries, each with their own unique functional attributes. To be sure, hadrosaurine and lambeosaurine hadrosaurids also differ in other aspects of tooth shape (e.g., curvature of primary carina, presence of secondary carinae, denticle coarseness, crown-root angle), but it is not immediately clear what adaptive significance—if any—these features might have had.

The pattern differs slightly when dental microwear is considered. Despite their different tooth morphologies, ankylosaurs cannot be differentiated from hadrosaurids (with the possible exception of nodosaurids and lambeosaurines), nor can ankylosaurids from nodosaurids. Interestingly, however, the hadrosaurids *Lambeosaurus* and *Prosaurolophus* are distinguishable according to their microwear fabrics, as may be centrosaurine and chasmosaurine ceratopsids. Thus, there possibly exist four distinguishable microwear types, but these do not necessarily correlate with tooth morphology.

Within the DPF, there were typically six megaherbivorous dinosaur species living in sympatry at any one time [10], comprising two ankylosaurs (one ankylosaurid plus one nodosaurid), two ceratopsids (one centrosaurine plus one chasmosaurine), and two hadrosaurids (one hadrosaurine plus one lambeosaurine). Therefore, considering evidence from either tooth morphology or wear in isolation, dietary niche partitioning cannot be invoked as the sole mechanism to explain the coexistence of these animals. On the other hand, if these two lines of evidence are considered in tandem, the niche partitioning hypothesis becomes more plausible because all sympatric taxa can be differentiated. Tooth morphology serves to discriminate the dietary ecology of the more inclusive taxa considered here, namely Ankylosauria, Ceratopsidae, and Hadrosauridae, in addition to the ankylosaur families Ankylosauridae and Nodosauridae. Dental microwear also distinguishes ceratopsids from ankylosaurs and hadrosaurids, and further discriminates the hadrosaurids *Lambeosaurus* and *Prosaurolophus*, and possibly centrosaurines and chasmosaurines. There is otherwise no particularly strong evidence for systematic dietary differences between Hadrosaurinae and Lambeosaurinae more generally, but the hadrosaurine sample remains small (comprising just four specimens of *Prosaurolophus* alone), and does not include *Gryposaurus* due to a lack of suitable material.

Given that unworn tooth morphology tells us only what a tooth *could* do, and that worn tooth morphology reflects what a tooth *did* do [34], [179], it is reasonable to ask if preference should be given to the microwear evidence. This may be so, but, given the necessarily limited microwear dataset used here, it is probably unwise to discount other types of evidence altogether. Recently, Fraser and Theodor [180] demonstrated that, while tooth wear dietary proxies reasonably distinguish ungulate browsers, grazers, and mixed feeders, a total evidence approach that includes morphological dietary proxies consistently yields the most accurate results.

Bearing this in mind, the case for niche partitioning among the megaherbivores from the DPF can be made even stronger if additional ecomorphologies are considered, including feeding height [11], skull [12] and beak morphology [13], and jaw mechanics [14]. These lines of evidence combine to strongly differentiate potential competitors, thereby increasing the likelihood of dietary niche partitioning, even among closely related taxa. Of course, it is possible that plant resources on Laramidia were not limiting and that sympatric herbivores shared the same diet, despite their morphological dissimilarities. However, this seems unlikely in light of the non-random replacement of inferred ecomorphotypes over the 1.5 Myr timespan of the DPF [10]. The finding that megaherbivorous dinosaurs from the DPF were limited by dietary resources suggests that they experienced the same bottom-up constraints as do living megaherbivorous mammals [3]–[5], which in turn imply common ecological limitations imposed by large body size.

Mallon and Anderson [12]–[14] and colleagues [10], [11] have argued that the megaherbivorous dinosaur assemblage of the DPF constitutes a chronofauna (sensu Olson [181]), based on the aforementioned temporal stability of ecomorphotypes. The present study lends additional support to this contention. The gradual decline of ankylosaurs from the upper half of the formation [10], [182], [183], however, suggests that perhaps woody browse was becoming increasingly more dominant across the coastal plain—something which these animals do not appear to have been particularly adept at eating. Corroborating this hypothesis, Eberth et al. [184] note that fossil tree trunks are most common in the upper 2/3 of the DPF.

## Conclusions

Evidence from tooth morphology and wear combine to support the hypothesis that dietary niche partitioning enabled the coexistence of megaherbivorous dinosaurs from the DPF. Gross tooth wear indicates that ankylosaurs were capable of feeding on tougher plant material than traditionally assumed, but they probably did not consume woody browse in as large quantities as ceratopsids and hadrosaurids. The larger, bladed teeth of nodosaurids suggest that these animals were better adapted to chewing more fibrous plants than ankylosaurids, although the limited microwear evidence available does not support this claim.

The complex dental batteries of ceratopsids and hadrosaurids divulge an affinity for particularly resistant plant tissues, but functional differences in the tooth arrangements imply related dietary differences. The strictly shearing ceratopsid dentition was best suited to rending the toughest plants, which likely included abundant leaf material. Microwear evidence further suggests that centrosaurines may have sustained themselves on a more abrasive diet than chasmosaurines, but other interpretations unrelated to diet are also possible. Conversely, the greater crushing component of the hadrosaurid dentition reveals that these animals were likely able to effectively masticate all types of plant parts, including leaves, fruits, seeds, and twigs. Dental microwear supports the contention that *Lambeosaurus* and *Prosaurolophus* fed on different plant tissues, but exactly what those were is not clear. These ecological relationships appear to have been stable over the 1.5 Myr duration of the DPF, as revealed by time-constrained analyses of dental microwear patterns. To the extent that the different megaherbivorous dinosaur families from the DPF are present in other Late Cretaceous fossil assemblages from Laramidia [8], [185], we anticipate that our findings are representative of those other assemblages also.

Tooth wear evidence also aids in the reconstruction of jaw mechanics. Ankylosaurs appear to have had an effectively propalinal power stroke, which contradicts traditional assumptions, but is otherwise in line with more recent work [66]. Ceratopsids and hadrosaurids were also capable of propaliny, but their power stroke was primarily directed orthopalinally, which accords with the findings of Varriale [42] and Williams et al. [27]. The occurrence of propaliny in ankylosaurs, ceratopsids, and hadrosaurids indicates that this was the ancestral condition for Genasauria.

This study is one of a small number of others [177], [186]–[190] to examine non-mammalian microwear in the context of palaeosynecology (sensu Ager [191]), and is the first to consider a Cretaceous community. The dearth of similar work serves to reinforce the basic need for further analyses of dinosaur microwear so that a better understanding of its variation can be gained. Given the fundamental differences between dinosaur and mammal teeth, it will never be possible to infer dinosaur palaeoecology using a strictly mammalian paradigm. For example, while major dietary categories (e.g., browser vs. grazer) of herbivorous mammals are generally best discriminated according to microwear scratch count [46], the more inclusive megaherbivorous dinosaur taxa are best discriminated using pit count. Nevertheless, it may be possible to discern dietary categories among dinosaurs if further effort is focused on elucidating both inter- and intraspecific variation in the microwear of these animals. This, in turn, will necessitate additional research into how tooth shape and replacement patterns influence microwear so that the dietary signal can be isolated. Furthermore, there is a need to learn more about how plants, particularly those that grew alongside the dinosaurs, produce microwear. Current understanding of how microwear forms stems largely from the study of mammals, particularly ungulates, which can be characterized as falling along a browser-grazer continuum [192], [193] or within a trophic triangle [50]. However, dinosaurs did not regularly consume grass, and so effort must be extended to understand the influence of various other plant types (e.g., ferns, cycads, horsetails, ginkgos, conifers) on microwear. Finally, it is necessary to examine microwear patterns in other Late Cretaceous assemblages of the North American Western Interior to determine whether the patterns recovered here are specific to the DPF, or whether they characterize the palaeoecology of Laramidia as a whole. This might be done most profitably with the aid of three-dimensional surface models and automated texture quantification, which minimize interobserver error [194]–[196].

## Supporting Information

Table S1 **Intact dentitions examined in this study.**
(DOCX)

Table S2 **Microwear data used in this study.**
(DOCX)

Table S3 **Wear features of isolated ankylosaur teeth from the Dinosaur Park Formation.**
(DOCX)

Table S4 **Microwear data for AMNH 5405 (** ***Euoplocephalus tutus*** **).**
(DOCX)

Table S5 **Microwear data for ROM 767 (** ***Centrosaurus apertus*** **).**
(DOCX)

Table S6 **Hadrosaurid microwear data comparing arcsine-transformed pit percentage (PP) and average vector length (r) between teeth in the lingual worn zone (LWZ) and buccal worn zone (BWZ).**
(DOCX)

Table S7 **Dental microwear data for CMN 2870 (** ***Prosaurolophus maximus*** **).**
(DOCX)
