# Supplementary material for: The Functional and Palaeoecological Implications of Tooth Morphology and Wear for the Megaherbivorous Dinosaurs from the Dinosaur Park Formation (Upper Campanian) of Alberta, Canada
Source: PLoS One. 2014 Jun 11;9(6):e98605. doi: 10.1371/journal.pone.0098605 (PMC4053334; doi:10.1371/journal.pone.0098605)
Supplement: Table S1 — Intact dentitions examined in this study. (DOCX) [file pone.0098605.s001.docx]

Table S1. Intact dentitions examined in this study.

| Suborder/family | Family/subfamily | Species | Specimen |
| --- | --- | --- | --- |
| Ankylosauria | Ankylosauridae | *Euoplocephalus tutus* | AMNH 5404 |
| Ankylosauria | Ankylosauridae | *Euoplocephalus tutus* | AMNH 5405 |
| Ankylosauria | Ankylosauridae | *Euoplocephalus tutus* | TMP 1997.132.0001 |
| Ankylosauria | Nodosauridae | *Panoplosaurus mirus* | ROM 1215 |
| Ankylosauria | Nodosauridae | *Panoplosaurus mirus* | TMP 1998.098.0001 |
| Ceratopsidae | Centrosaurinae | *Centrosaurus apertus* | AMNH 5237 |
| Ceratopsidae | Centrosaurinae | *Centrosaurus apertus* | AMNH 5351 |
| Ceratopsidae | Centrosaurinae | *Centrosaurus apertus* | AMNH 5377 |
| Ceratopsidae | Centrosaurinae | *Centrosaurus apertus* | CMN 348 |
| Ceratopsidae | Centrosaurinae | *Centrosaurus apertus* | CMN 8795 |
| Ceratopsidae | Centrosaurinae | *Centrosaurus apertus* | ROM 767 |
| Ceratopsidae | Centrosaurinae | *Centrosaurus apertus* | TMP 1997.085.0001 |
| Ceratopsidae | Centrosaurinae | *Centrosaurus apertus* | UALVP 16248 |
| Ceratopsidae | Centrosaurinae | *Centrosaurus apertus* | UALVP 41 |
| Ceratopsidae | Centrosaurinae | *Centrosaurus apertus* | USNM 8897 |
| Ceratopsidae | Centrosaurinae | *Centrosaurus apertus* | YPM 2015 |
| Ceratopsidae | Centrosaurinae | ‘pachyrhinosaur’ | TMP 2002.076.0001 |
| Ceratopsidae | Centrosaurinae | *Styracosaurus albertensis* | CMN 344 |
| Ceratopsidae | Centrosaurinae | *Styracosaurus albertensis* | TMP 1986.126.0001 |
| Ceratopsidae | Chasmosaurinae | *Chasmosaurus belli* | CMN 2245 |
| Ceratopsidae | Chasmosaurinae | *Chasmosaurus belli* | ROM 839 |
| Ceratopsidae | Chasmosaurinae | *Chasmosaurus belli* | ROM 843 |
| Ceratopsidae | Chasmosaurinae | *Chasmosaurus russelli* | CMN 2280 |
| Ceratopsidae | Chasmosaurinae | *Chasmosaurus russelli* | TMP 1981.019.0175 |
| Ceratopsidae | Chasmosaurinae | *Chasmosaurus sp.* | CMN 8801 |
| Ceratopsidae | Chasmosaurinae | *Vagaceratops irvinensis* | CMN 41357 |
| Hadrosauridae | Hadrosaurinae | *Gryposaurus notabilis* | CMN 2278 |
| Hadrosauridae | Hadrosaurinae | *Gryposaurus notabilis* | ROM 764 |
| Hadrosauridae | Hadrosaurinae | *Gryposaurus notabilis* | ROM 873 |
| Hadrosauridae | Hadrosaurinae | *Gryposaurus notabilis* | TMP 1980.022.0001 |
| Hadrosauridae | Hadrosaurinae | *Gryposaurus notabilis* | TMP 1991.081.0001 |
| Hadrosauridae | Hadrosaurinae | *Prosaurolophus maximus* | CMN 2277 |
| Hadrosauridae | Hadrosaurinae | *Prosaurolophus maximus* | CMN 2870 |
| Hadrosauridae | Hadrosaurinae | *Prosaurolophus maximus* | CMN 8894 |
| Hadrosauridae | Hadrosaurinae | *Prosaurolophus maximus* | ROM 1928 |
| Hadrosauridae | Hadrosaurinae | *Prosaurolophus maximus* | ROM 787 |
| Hadrosauridae | Hadrosaurinae | *Prosaurolophus maximus* | TMM 41262 |
| Hadrosauridae | Hadrosaurinae | *Prosaurolophus maximus* | TMP 1984.001.0001 |
| Hadrosauridae | Hadrosaurinae | *Prosaurolophus maximus* | USNM 12712 |
| Hadrosauridae | Lambeosaurinae | *Corythosaurus casuarius* | ROM 870 |
| Hadrosauridae | Lambeosaurinae | *Corythosaurus casuarius* | ROM 871 |
| Hadrosauridae | Lambeosaurinae | *Corythosaurus casuarius* | TMP 1980.040.0001 |
| Hadrosauridae | Lambeosaurinae | *Corythosaurus casuarius* | TMP 1984.121.0001 |
| Hadrosauridae | Lambeosaurinae | *Corythosaurus intermedius* | CMN 8676 |
| Hadrosauridae | Lambeosaurinae | *Corythosaurus intermedius* | CMN 8704 |
| Hadrosauridae | Lambeosaurinae | *Corythosaurus intermedius* | ROM 776 |
| Hadrosauridae | Lambeosaurinae | *Corythosaurus intermedius* | ROM 777 |
| Hadrosauridae | Lambeosaurinae | *Corythosaurus intermedius* | ROM 845 |
| Hadrosauridae | Lambeosaurinae | *Corythosaurus intermedius* | TMP 1980.023.0004 |
| Hadrosauridae | Lambeosaurinae | *Corythosaurus* sp. | CMN 34825 |
| Hadrosauridae | Lambeosaurinae | *Corythosaurus* sp. | FMNH 1357 |
| Hadrosauridae | Lambeosaurinae | *Corythosaurus* sp. | ROM 1947 |
| Hadrosauridae | Lambeosaurinae | *Corythosaurus* sp. | ROM 759 |
| Hadrosauridae | Lambeosaurinae | *Corythosaurus* sp. | ROM 868 |
| Hadrosauridae | Lambeosaurinae | *Corythosaurus* sp. | TMP 1982.037.0001 |
| Hadrosauridae | Lambeosaurinae | *Corythosaurus* sp. | TMP 1997.012.0232 |
| Hadrosauridae | Lambeosaurinae | *Lambeosaurus clavinitialis* | AMNH 5382 |
| Hadrosauridae | Lambeosaurinae | *Lambeosaurus clavinitialis* | CMN 8703 |
| Hadrosauridae | Lambeosaurinae | *Lambeosaurus clavinitialis* | ROM 869 |
| Hadrosauridae | Lambeosaurinae | *Lambeosaurus clavinitialis* | TMP 1981.037.0001 |
| Hadrosauridae | Lambeosaurinae | *Lambeosaurus* *clavinitialis* | YPM 3222 |
| Hadrosauridae | Lambeosaurinae | *Lambeosaurus lambei* | CMN 2869 |
| Hadrosauridae | Lambeosaurinae | *Lambeosaurus lambei* | FMNH 1479 |
| Hadrosauridae | Lambeosaurinae | *Lambeosaurus lambei* | ROM 1218 |
| Hadrosauridae | Lambeosaurinae | *Lambeosaurus lambei* | ROM 794 |
| Hadrosauridae | Lambeosaurinae | *Lambeosaurus lambei* | TMP 1982.038.0001 |
| Hadrosauridae | Lambeosaurinae | *Lambeosaurus lambei* | TMP 1997.012.0128 |
| Hadrosauridae | Lambeosaurinae | *Lambeosaurus magnicristatus* | CMN 8705 |
| Hadrosauridae | Lambeosaurinae | *Lambeosaurus magnicristatus* | TMP 1966.0004.0001 |
| Hadrosauridae | Lambeosaurinae | *Lambeosaurus* sp. | CMN 351 |
| Hadrosauridae | Lambeosaurinae | *Lambeosaurus* sp. | CMN 8503 |
| Hadrosauridae | Lambeosaurinae | *Lambeosaurus* sp. | CMN 8633 |
| Hadrosauridae | Lambeosaurinae | *Lambeosaurus* sp. | NHMUK R9527 |
| Hadrosauridae | Lambeosaurinae | *Lambeosaurus* sp. | ROM 758 |
| Hadrosauridae | Lambeosaurinae | *Lambeosaurus* sp. | USNM 10309 |
| Hadrosauridae | Lambeosaurinae | *Parasaurolophus walkeri* | ROM 768 |
