# Supplementary material for: The Functional and Palaeoecological Implications of Tooth Morphology and Wear for the Megaherbivorous Dinosaurs from the Dinosaur Park Formation (Upper Campanian) of Alberta, Canada
Source: PLoS One. 2014 Jun 11;9(6):e98605. doi: 10.1371/journal.pone.0098605 (PMC4053334; doi:10.1371/journal.pone.0098605)
Supplement: Table S2 — Microwear data used in this study. (DOCX) [file pone.0098605.s002.docx]

Table S2. Microwear data used in this study. Abbreviations: MAZ, Megaherbivore Assemblage Zone; S, average scratch count; P, average pit count; W, average feature width.

| Suborder/family | Family/subfamily | Genus | Specimen | S | P | W (μm) | MAZ |
| --- | --- | --- | --- | --- | --- | --- | --- |
| Ankylosauria | Ankylosauridae | ? | TMP 1986.198.0003 | 24.00 | 3.00 | 17.56 | 1 |
| Ankylosauria | Ankylosauridae | ? | TMP 1987.157.0035 | 24.00 | 1.50 | 23.53 | 1 |
| Ankylosauria | Ankylosauridae | ? | TMP 1991.050.0014 | 68.50 | 6.50 | 12.17 | ? |
| Ankylosauria | Ankylosauridae | ? | TMP 1997.012.0042 | 35.00 | 4.00 | 15.41 | ? |
| Ankylosauria | Ankylosauridae | ? | TMP 1997.012.0072 | 26.00 | 1.00 | 16.67 | ? |
| Ankylosauria | Ankylosauridae | ? | TMP 1997.012.0106 | 56.00 | 4.50 | 14.72 | ? |
| Ankylosauria | Ankylosauridae | *Euoplocephalus* | AMNH 5238 | 34.00 | 4.00 | 16.01 | ? |
| Ankylosauria | Ankylosauridae | *Euoplocephalus* | AMNH 5404 | 50.88 | 10.88 | 13.45 | 1 |
| Ankylosauria | Ankylosauridae | *Euoplocephalus* | AMNH 5405 | 46.79 | 5.07 | 13.52 | ? |
| Ankylosauria | Nodosauridae | ? | TMP 1986.008.0081 | 23.00 | 5.00 | 23.32 | 2 |
| Ankylosauria | Nodosauridae | ? | TMP 1986.010.0071 | 25.00 | 3.00 | 26.18 | 1 |
| Ankylosauria | Nodosauridae | ? | TMP 1986.023.0108 | 36.00 | 8.00 | 16.82 | 1 |
| Ankylosauria | Nodosauridae | ? | TMP 1991.050.0126 | 64.00 | 5.50 | 11.76 | ? |
| Ankylosauria | Nodosauridae | ? | TMP 1994.012.0104 | 40.50 | 4.00 | 13.25 | 1 |
| Ankylosauria | Nodosauridae | ? | TMP 1994.012.0229 | 36.50 | 3.00 | 14.59 | 1 |
| Ankylosauria | Nodosauridae | ? | TMP 1995.012.0085 | 40.00 | 9.00 | 14.16 | ? |
| Ankylosauria | Nodosauridae | ? | TMP 1995.147.0029 | 40.00 | 4.00 | 11.58 | 1 |
| Ankylosauria | Nodosauridae | ? | TMP 2000.012.0024 | 61.50 | 6.50 | 10.10 | ? |
| Ankylosauria | Nodosauridae | *Panoplosaurus* | AMNH 5381 | 45.00 | 5.00 | 14.01 | 1 |
| Ankylosauria | Nodosauridae | *Panoplosaurus* | ROM 1215 | 60.88 | 9.63 | 13.93 | 1 |
| Ceratopsidae | Centrosaurinae | *Centrosaurus* | AMNH 5237 | 56.17 | 3.50 | 12.93 | ? |
| Ceratopsidae | Centrosaurinae | *Centrosaurus* | ROM 767 | 39.58 | 1.67 | 13.89 | 1 |
| Ceratopsidae | Centrosaurinae | *Centrosaurus* | TMP 1997.085.0001 | 45.38 | 1.38 | 13.15 | 1 |
| Ceratopsidae | Centrosaurinae | *Centrosaurus* | UALVP 41 | 46.75 | 2.25 | 11.96 | ? |
| Ceratopsidae | Centrosaurinae | *Centrosaurus* | UALVP 16248 | 40.50 | 2.30 | 15.44 | 1 |
| Ceratopsidae | Centrosaurinae | ‘pachyrhinosaur’ | TMP 2002.076.0001 | 41.50 | 1.50 | 15.35 | 2 |
| Ceratopsidae | Centrosaurinae | *Styracosaurus* | CMN 344 | 36.00 | 1.00 | 14.26 | 2 |
| Ceratopsidae | Chasmosaurinae | *Chasmosaurus* | CMN 8801 | 42.50 | 1.00 | 13.17 | 1 |
| Ceratopsidae | Chasmosaurinae | *Chasmosaurus* | CMN 8802 | 19.00 | 0.50 | 21.56 | 1 |
| Ceratopsidae | Chasmosaurinae | *Chasmosaurus* | ROM 839 | 32.60 | 0.80 | 14.13 | 2 |
| Ceratopsidae | Chasmosaurinae | *Chasmosaurus* | ROM 843 | 39.00 | 2.00 | 12.41 | 2 |
| Ceratopsidae | Chasmosaurinae | *Vagaceratops* | CMN 41357 | 28.83 | 2.50 | 14.44 | 2 |
| Hadrosauridae | Hadrosaurinae | *Prosaurolophus* | CMN 2870 | 37.46 | 3.29 | 14.70 | ? |
| Hadrosauridae | Hadrosaurinae | *Prosaurolophus* | ROM 1928 | 59.75 | 5.88 | 11.37 | 2 |
| Hadrosauridae | Hadrosaurinae | *Prosaurolophus* | ROM 787 | 39.75 | 3.00 | 13.87 | 2 |
| Hadrosauridae | Hadrosaurinae | *Prosaurolophus* | TMM 41262 | 56.17 | 3.17 | 12.36 | 2 |
| Hadrosauridae | Lambeosaurinae | *Corythosaurus* | CMN 34825 | 35.92 | 3.42 | 16.18 | 1 |
| Hadrosauridae | Lambeosaurinae | *Corythosaurus* | ROM 1933 | 43.75 | 8.75 | 12.31 | 1 |
| Hadrosauridae | Lambeosaurinae | *Corythosaurus* | ROM 1947 | 47.17 | 4.67 | 13.19 | 1 |
| Hadrosauridae | Lambeosaurinae | *Corythosaurus* | ROM 868 | 35.75 | 1.75 | 13.92 | 1 |
| Hadrosauridae | Lambeosaurinae | *Corythosaurus* | ROM 871 | 33.29 | 2.50 | 17.05 | 1 |
| Hadrosauridae | Lambeosaurinae | *Corythosaurus* | TMP 1982.037.0001 | 58.22 | 4.41 | 12.36 | 1 |
| Hadrosauridae | Lambeosaurinae | *Corythosaurus* | TMP 1997.012.0232 | 37.63 | 3.19 | 14.08 | ? |
| Hadrosauridae | Lambeosaurinae | *Lambeosaurus* | CMN 2869 | 31.00 | 1.80 | 14.87 | ? |
| Hadrosauridae | Lambeosaurinae | *Lambeosaurus* | CMN 8503 | 36.70 | 2.00 | 14.21 | 2 |
| Hadrosauridae | Lambeosaurinae | *Lambeosaurus* | CMN 8633 | 28.67 | 2.33 | 15.08 | 1 |
| Hadrosauridae | Lambeosaurinae | *Lambeosaurus* | CMN 8703 | 38.25 | 4.91 | 14.99 | 1 |
| Hadrosauridae | Lambeosaurinae | *Lambeosaurus* | ROM 794 | 40.86 | 9.36 | 14.20 | 2 |
| Hadrosauridae | Lambeosaurinae | *Lambeosaurus* | TMP 1981.037.0001 | 32.75 | 4.08 | 18.69 | 1 |
| Hadrosauridae | Lambeosaurinae | *Lambeosaurus* | USNM 10309 | 28.00 | 3.50 | 17.98 | ? |
| Hadrosauridae | Lambeosaurinae | *Lambeosaurus* | YPM 3222 | 32.25 | 3.00 | 16.42 | 2 |
