# Supplementary material for: The Functional and Palaeoecological Implications of Tooth Morphology and Wear for the Megaherbivorous Dinosaurs from the Dinosaur Park Formation (Upper Campanian) of Alberta, Canada
Source: PLoS One. 2014 Jun 11;9(6):e98605. doi: 10.1371/journal.pone.0098605 (PMC4053334; doi:10.1371/journal.pone.0098605)
Supplement: Table S3 — Wear features of isolated ankylosaur teeth from the Dinosaur Park Formation. (DOCX) [file pone.0098605.s003.docx]

Table S3. Wear features of isolated ankylosaur teeth from the Dinosaur Park Formation. ‘Paired facets’ refers to the presence of mesiodistally arranged wear facets on a single tooth surface (labial or lingual). ‘IPF’ refers to the presence of an ‘interdental pressure’ facet (sensu Thulborn [1])

| Family | Specimen (TMP) | Facet count | Facet inclination | Paired facets? | IPF? |
| --- | --- | --- | --- | --- | --- |
| Ankylosauridae | 1981.016.0477 | 2 | vertical | no | no |
| Ankylosauridae | 1992.036.1178 | 1 | vertical | -- | no |
| Ankylosauridae | 1993.079.0066 | 1 | oblique | -- | no |
| Ankylosauridae | 1994.094.0014b | 1 | vertical | -- | no |
| Ankylosauridae | 1997.012.0042 | 1 | vertical | -- | no |
| Ankylosauridae | 1997.012.0072 | 2 | oblique/vertical | no | no |
| Ankylosauridae | 1997.012.0102 | 2 | horizontal/vertical | no | no |
| Ankylosauridae | 1997.012.0106 | 2 | oblique | no | no |
| Ankylosauridae | 1999.055.0081 | 1 | oblique | -- | no |
| Ankylosauridae | 1999.055.0162 | 1 | vertical | -- | no |
| Ankylosauridae | 1999.055.0246 | 1 | oblique | -- | no |
| Ankylosauridae | 1999.055.0290 | 1 | vertical | -- | no |
| Ankylosauridae | 2001.012.0073 | 2 | oblique/vertical | no | no |
| Ankylosauridae | 2001.012.0074 | 1 | vertical | -- | no |
| Ankylosauridae | 2002.012.0060 | 2 | vertical | no | no |
| Ankylosauridae | 2005.012.0233 | 1 | vertical | -- | no |
| Ankylosauridae | 2005.012.0384a | 1 | oblique | -- | no |
| Ankylosauridae | 2005.012.0384b | 2 | vertical | no | no |
| Ankylosauridae | 2011.047.0079 | 1 | oblique | -- | no |
| Nodosauridae | 1992.030.0231 | 1 | vertical | -- | no |
| Nodosauridae | 1993.036.0081 | 1 | vertical | -- | no |
| Nodosauridae | 1993.036.0458 | 1 | oblique | -- | no |
| Nodosauridae | 1993.036.0484 | 1 | oblique | -- | no |
| Nodosauridae | 1994.012.0029 | 1 | oblique | -- | no |
| Nodosauridae | 1994.012.0035 | 1 | oblique | -- | no |
| Nodosauridae | 1994.016.0005 | 1 | oblique | -- | no |
| Nodosauridae | 1994.012.0076 | 1 | oblique | -- | no |
| Nodosauridae | 1994.012.0077 | 1 | oblique | -- | no |
| Nodosauridae | 1994.012.0104a | 1 | vertical | -- | no |
| Nodosauridae | 1994.012.0104b | 1 | oblique | -- | no |
| Nodosauridae | 1994.012.0104c | 1 | oblique | -- | no |
| Nodosauridae | 1994.012.0120 | 1 | oblique | -- | no |
| Nodosauridae | 1994.012.0194 | 1 | oblique | -- | no |
| Nodosauridae | 1994.012.0229a | 1 | oblique | -- | no |
| Nodosauridae | 1994.012.0229b | 1 | oblique | -- | no |
| Nodosauridae | 1994.012.0252b | 1 | oblique | -- | no |
| Nodosauridae | 1994.012.0252c | 1 | oblique | -- | no |
| Nodosauridae | 1994.012.0447 | 1 | oblique | -- | no |
| Nodosauridae | 1994.094.0014a | 1 | oblique | -- | no |
| Nodosauridae | 1994.172.0045 | 1 | oblique | -- | no |
| Nodosauridae | 1995.012.0029 | 1 | oblique | -- | no |
| Nodosauridae | 1995.012.0085 | 3 | horizontal/vertical | no | yes |
| Nodosauridae | 1995.012.0117 | 2 | oblique/vertical | yes | no |
| Nodosauridae | 1995.179.0001a | 1 | oblique | -- | no |
| Nodosauridae | 1995.179.0001b | 1 | oblique | -- | no |
| Nodosauridae | 1997.012.0005 | 2 | vertical | yes | no |
| Nodosauridae | 1997.012.0041b | 1 | oblique | -- | no |
| Nodosauridae | 1997.012.0041c | 1 | oblique | -- | no |
| Nodosauridae | 1997.012.0041a | 2 | oblique/vertical | no | no |
| Nodosauridae | 1997.012.0085 | 1 | oblique | -- | no |
| Nodosauridae | 1998.068.0080 | 1 | oblique | -- | no |
| Nodosauridae | 1998.068.0087 | 2 | oblique/vertical | no | no |
| Nodosauridae | 1998.068.0120 | 1 | vertical | -- | no |
| Nodosauridae | 1998.068.0141 | 1 | oblique | -- | no |
| Nodosauridae | 1998.068.0153 | 1 | vertical | -- | no |
| Nodosauridae | 1999.063.0019 | 1 | vertical | -- | no |
| Nodosauridae | 1999.055.0243 | 1 | horizontal | -- | no |
| Nodosauridae | 1999.085.0005 | 1 | vertical | -- | no |
| Nodosauridae | 1999.085.0012 | 2 | horizontal/vertical | no | no |
| Nodosauridae | 2000.012.0026 | 1 | oblique | -- | no |
| Nodosauridae | 2000.012.0028 | 4 | horizontal/oblique/vertical | no | yes |
| Nodosauridae | 2000.057.0014a | 1 | oblique | -- | no |
| Nodosauridae | 2000.057.0014b | 1 | horizontal | -- | no |
| Nodosauridae | 2000.057.0052 | 1 | oblique | -- | no |
| Nodosauridae | 2000.057.0067 | 1 | oblique | -- | no |
| Nodosauridae | 2002.060.0002e | 1 | oblique | -- | no |
| Nodosauridae | 2002.060.0002a | 1 | vertical | -- | no |
| Nodosauridae | 2002.060.0002b | 1 | vertical | -- | no |
| Nodosauridae | 2002.060.0002c | 1 | oblique | -- | no |
| Nodosauridae | 2002.060.0002f | 1 | oblique | -- | no |
| Nodosauridae | 2002.060.0002d | 2 | oblique/vertical | no | no |
| Nodosauridae | 2003.012.0259 | 1 | oblique | -- | no |
| Nodosauridae | 2003.012.0285 | 1 | horizontal | -- | no |
| Nodosauridae | 2004.103.0012 | 1 | horizontal | -- | no |
| Nodosauridae | 2004.104.0012 | 1 | oblique | -- | no |
| Nodosauridae | 2004.110.0014 | 1 | oblique | -- | no |
| Nodosauridae | 2004.114.0004 | 1 | oblique | -- | no |
| Nodosauridae | 2004.116.0019 | 1 | oblique | -- | no |
| Nodosauridae | 2004.116.0036 | 1 | oblique | -- | no |
| Nodosauridae | 2005.012.0132 | 1 | oblique | -- | no |
| Nodosauridae | 2005.012.0185 | 1 | oblique | -- | no |
| Nodosauridae | 2005.012.0232b | 1 | oblique | -- | no |
| Nodosauridae | 2005.012.0232a | 2 | horizontal/vertical | no | no |
| Nodosauridae | 2005.012.0279 | 1 | vertical | -- | no |
| Nodosauridae | 2005.012.0280 | 1 | oblique | -- | no |
| Nodosauridae | 2005.012.0368a | 1 | vertical | -- | no |
| Nodosauridae | 2005.012.0368b | 1 | oblique | -- | no |
| Nodosauridae | 2005.012.0368c | 1 | vertical | -- | no |
| Nodosauridae | 2005.012.0368d | 1 | oblique | -- | no |
| Nodosauridae | 2005.012.0380 | 1 | oblique | -- | no |
| Nodosauridae | 2005.012.0397 | 1 | vertical | -- | no |
| Nodosauridae | 2005.054.0007 | 1 | oblique | -- | no |
| Nodosauridae | 2005.012.0496 | 1 | vertical | -- | no |
| Nodosauridae | 2005.049.0127 | 1 | oblique | -- | no |
| Nodosauridae | 2005.049.0142 | 1 | oblique | -- | no |
| Nodosauridae | 2007.020.0042 | 1 | oblique | -- | no |
| Nodosauridae | 2011.012.0027 | 1 | oblique | -- | no |
| Nodosauridae | 2011.047.0010 | 1 | vertical | -- | no |

**Literature cited**

1. Thulborn RA (1974) Thegosis in herbivorous dinosaurs. Nature 250: 729–731.
