# Supplementary material for: The Functional and Palaeoecological Implications of Tooth Morphology and Wear for the Megaherbivorous Dinosaurs from the Dinosaur Park Formation (Upper Campanian) of Alberta, Canada
Source: PLoS One. 2014 Jun 11;9(6):e98605. doi: 10.1371/journal.pone.0098605 (PMC4053334; doi:10.1371/journal.pone.0098605)
Supplement: Table S4 — Microwear data for AMNH 5405 ( Euoplocephalus tutus ). (DOCX) [file pone.0098605.s004.docx]

Table S4. Microwear data for AMNH 5405 (*Euoplocephalus tutus*). Abbreviations: S, scratch count; P, pit count; W, average feature width.

| Tooth position | S | P | W (μm) |
| --- | --- | --- | --- |
| RM 1 | 49 | 11 | 10.85 |
| RM 3 | 93 | 10 | 14.83 |
| LM 5 | 97 | 5 | 12.34 |
| LM 9 | 79 | 5 | 14.05 |
| RM 14 | 113 | 16 | 13.16 |
| RM 16 | 81 | 7 | 15.26 |
| RM 17 | 47 | 3 | 14.18 |
