# Supplementary material for: The Functional and Palaeoecological Implications of Tooth Morphology and Wear for the Megaherbivorous Dinosaurs from the Dinosaur Park Formation (Upper Campanian) of Alberta, Canada
Source: PLoS One. 2014 Jun 11;9(6):e98605. doi: 10.1371/journal.pone.0098605 (PMC4053334; doi:10.1371/journal.pone.0098605)
Supplement: Table S5 — Microwear data for ROM 767 ( Centrosaurus apertus ). (DOCX) [file pone.0098605.s005.docx]

Table S5. Microwear data for ROM 767 (*Centrosaurus apertus*). Abbreviations: S, scratch count; P, pit count; W, average feature width.

| Tooth position | S | P | W (μm) |
| --- | --- | --- | --- |
| RD 13 | 26 | 0 | 14.42 |
| RD 14 | 56.5 | 2 | 10.79 |
| RD 15 | 57.5 | 0 | 9.70 |
| RD 17 | 41 | 3 | 14.00 |
| RD 18 | 18 | 1 | 21.95 |
| RD 19 | 38.5 | 4 | 12.51 |
