# Supplementary material for: The Functional and Palaeoecological Implications of Tooth Morphology and Wear for the Megaherbivorous Dinosaurs from the Dinosaur Park Formation (Upper Campanian) of Alberta, Canada
Source: PLoS One. 2014 Jun 11;9(6):e98605. doi: 10.1371/journal.pone.0098605 (PMC4053334; doi:10.1371/journal.pone.0098605)
Supplement: Table S6 — Hadrosaurid microwear data comparing arcsine-transformed pit percentage (PP) and average vector length (r) between teeth in the lingual worn zone (LWZ) and buccal worn zone (BWZ). (DOCX) [file pone.0098605.s006.docx]

Table S6. Hadrosaurid microwear data comparing arcsine-transformed pit percentage (PP) and average vector length (r) between teeth in the lingual worn zone (LWZ) and buccal worn zone (BWZ).

|  | LWZ | | | BWZ | | |
| --- | --- | --- | --- | --- | --- | --- |
| Specimen | Tooth position | Arcsine PP | r | Tooth position | Arcsine PP | r |
| ROM 868 | LD 19(1) | 13.76 | 0.54 | LD 17(3) | 15.50 | 0.45 |
| ROM 868 | LD 18(2) | 20.70 | 0.49 | LD 18(3) | 21.52 | 0.58 |
| ROM 868 | LD 21(2) | 0 | 0.35 | LD 21(3) | 0 | 0.29 |
| TMP 1982.037.0001 | LD 12(1) | 13.93 | 0.18 | LD 11(3) | 14.96 | 0.66 |
| TMP 1982.037.0001 | RD 32(2) | 8.95 | 0.31 | RD 32(3) | 8.48 | 0.23 |
| ROM 794 | RD 24(1) | 25.98 | 0.43 | RD 25(3) | 24.46 | 0.37 |
| YPM 3222 | LD 26(2) | 19.61 | 0.34 | LD 26(3) | 26.57 | 0.63 |
