# Supplementary material for: The Functional and Palaeoecological Implications of Tooth Morphology and Wear for the Megaherbivorous Dinosaurs from the Dinosaur Park Formation (Upper Campanian) of Alberta, Canada
Source: PLoS One. 2014 Jun 11;9(6):e98605. doi: 10.1371/journal.pone.0098605 (PMC4053334; doi:10.1371/journal.pone.0098605)
Supplement: Table S7 — Dental microwear data for CMN 2870 ( Prosaurolophus maximus ). (DOCX) [file pone.0098605.s007.docx]

Table S7. Dental microwear data for CMN 2870 (*Prosaurolophus maximus*). Abbreviations: S, scratch count; P, pit count; W, average feature width

| Tooth position | S | P | W (μm) |
| --- | --- | --- | --- |
| LD 2 | 13.5 | 5.5 | 24.18 |
| LD 3 | 27.5 | 1.5 | 19.52 |
| LD 4 | 26.5 | 4.5 | 17.26 |
| LD 5 | 45 | 2 | 13.72 |
| LD 8 | 32 | 6 | 12.76 |
| LD 9 | 28.5 | 4.5 | 16.05 |
| LD 11 | 33 | 4 | 14.61 |
| RD 12 | 39.5 | 9.5 | 13.84 |
| RD 13 | 46.5 | 2.5 | 12.40 |
| RD 14 | 42 | 3.5 | 13.80 |
| LD 15 | 33.5 | 2 | 14.41 |
| RD 16 | 42.5 | 1.5 | 14.31 |
| RD 17 | 40.5 | 2 | 12.72 |
| RD 18 | 34 | 2 | 15.88 |
| RD 19 | 40.5 | 4 | 13.91 |
| RD 20 | 33 | 1 | 15.69 |
| RD 21 | 37 | 3.5 | 14.37 |
| RD 22 | 45 | 2 | 12.81 |
| RD 23 | 50.5 | 1 | 12.40 |
| RD 24 | 44.5 | 3.5 | 13.13 |
| RD 25 | 39 | 1 | 16.94 |
| LD 26 | 36 | 5.5 | 11.90 |
| RD 27 | 51.5 | 2 | 14.07 |
| LD 28 | 37.5 | 4.5 | 12.18 |
